# Basal metabolic rate and risk for diabetes and its complications among 341,790 adults from the UK Biobank

**DOI:** 10.1016/j.diabres.2026.113198

**Published:** 2026-05

**Authors:** Joseph Frimpong, Andrew Browne, Wrivu Martin, Pallavi Kaushik, Louisa Gnatiuc Friedrichs

**Affiliations:** Clinical Trial Service Unit & Epidemiological Studies Unit, Nuffield Department of Population Health, University of Oxford, UK

**Keywords:** Diabetes, Metabolic rate, Energy balance, Cohort study, UK biobank

## Abstract

**Aims:**

Basal metabolic rate (BMR) and energy imbalance are determinants of diabetes. We assessed the BMR-associated risk for diabetes in a large cohort.

**Methods:**

Cox proportional models estimated hazard ratios (HR) for subsequent diabetes and its complications with estimated BMR among diabetes-free participants. The mediating role of vascular-metabolic biomarkers were assessed.

**Findings:**

Among 341,790 participants aged 55.7 ± 8.1 years, the mean (standard deviation [SD]) estimated BMR was 1849 (234) kcal/day in men and 1340 (147) kcal/day in women. Over 12 years follow-up, 4,626 cases of diabetes accrued. Higher estimated BMR showed positive associations with diabetes, with each SD higher estimated BMR associated with a 55% increase in diabetes risk (HR 1.54, 95% confidence interval [CI] 1.49–1.59 in men; HR 1.56, 1.49–1.62 in women), and a substantive risk for cardiac complications among those with diabetes (1.71, 1.55–1.89 in men; 1.78, 1.56–2.03 in women), diabetic coma, ketoacidosis and glycaemic disturbances (1.18, 0.99–1.42 in men; 1.35, 1.21–1.50 in women), and stroke (1.27, 1.08–1.50 in men; 1.41, 1.14–1.76 in women). Glycated haemoglobin, triglycerides, high-density-cholesterol, Cystatin-C and C-reactive protein explained 10–30% of these associations.

**Conclusion:**

Estimated elevated BMR was strongly positively associated with risk for diabetes and its complications, suggesting to re-evaluate the role of BMR, in addition to BMI, in diabetes research.

## Highlights

• To date, there was conflicting evidence of genetically-predicted BMR on risk for type 2 DM and limited large prospective data.
• This study of  > 340,000 adults, estimated a 55% higher risk for subsequent DM with bio-impedance estimated BMR at ages 35–75 years.
• The DM risk was about half as strong in non-Caucasians compared to Caucasians.
• The risk for DM complications appeared stronger in women: a 78% greater risk for cardiac events, a 35% greater risk for diabetic coma, ketoacidosis and glycaemic disturbances and a 41% greater risk for stroke.
• Up to 30% of the risk was mediated by glycated haemoglobin, triglycerides, high-density cholesterol, Cystatin-C and C-reactive protein.

## 1 Background

Diabetes has emerged as an increasing major global health priority. In 2021, 537 million adults were estimated to have diabetes globally, with projections suggesting a rise to 643 million by 2030 and to 784 million by 2045 [1]. Type 2 diabetes mellitus (T2DM), the most common type of diabetes, is known to be largely driven by genetic, environmental, lifestyle and biological factors, including obesity, dyslipidaemia and glycaemic imbalance. Excess adiposity is a major preventable determinant of diabetes risk, and is largely driven by long-term energy imbalance between energy intake and energy expenditure [2]. For instance, high consumption of energy-dense foods and insufficient physical activity may lead to a positive energy balance, which promotes excess adiposity and insulin resistance, both of which are key mechanisms in the pathogenesis of T2DM [3], [4]. However, these behavioural risk factors affect only partially the energy balance equation. Basal metabolic rate (BMR), the energy required for maintaining essential physiological functions, including cellular repair, circulation, and respiration when at rest and in a post-absorptive state, [5] accounts for the largest share (∼60–70%) of daily energy expenditure [6]. As a largely physiologically determined and relatively stable component of energy expenditure, BMR may play an underappreciated role in regulating energy balance and consequent risk for diabetes. Previous studies have linked low BMR to weight gain and insulin resistance, [7], [8] suggesting that BMR may influence diabetes risk independent of excess weight. However, large population-level evidence supporting the association of BMR with T2DM remains limited and inconclusive. Although previous studies have described elevated rather than low levels of BMR in subjects with T2DM, [9], [10], [11] the relatively small sample sizes (<500 individuals), cross-sectional [10] or case-control design [9], [11] increases the potential for spurious findings and biases such as reverse causality. Two-sample Mendelian Randomisation (MR) studies have investigated the causal effect of BMR for T2DM, however, the results have been inconsistent, and they did not account for the relevance of BMR independent of genetic liability to BMI. One MR study reported a 49% higher odds of T2DM with increase in genetically determined BMR, [12] the second reported null findings [13]. Clarifying these associations may offer new insights into diabetes pathophysiology, aid identifying high-risk individuals, and enable early personalised preventive interventions to prevent diabetes and its complications. The UK Biobank, offers a unique opportunity to reliably investigate these hypotheses. We aimed to assess the prospective association between BMR and subsequent risk of diabetes, including diabetes subtypes and related complications, in one of the largest UK population cohorts.

## 2 Methods

### 2.1 Study design and data collection

The design and methodology of the UK Biobank have been detailed elsewhere [14], [15]. Briefly, between 2006 and 2010, individuals aged 40–69 years and residing within 25 miles of an assessment centre, were invited to participate. Of these, 503,317 adults consented and attended one of 22 research assessment centres across the UK [15]. During the first visit, detailed baseline information on socio-demographic, lifestyle, medical and other health-related factors were collected via a self-administered computer touchscreen questionnaire. Trained research staff took anthropometric and other physical measurements and collected blood samples. Resurvey visits re-assessed the general, health-related and biological characteristics of a subset of the cohort, by using protocols similar to baseline. Indefinite follow-up for hospital events or deaths is conducted in the entire cohort regularly and medical causes are recorded.

Ethical approval was obtained from the North West Multi-Centre Research Ethics Committee (Ref 11/NW/0382 on June 17, 2011), and all participants provided written informed consent to participate.

### 2.2 Basal metabolic rate measurement

Basal metabolic rate was estimated among 492,011 participants at recruitment, along whole-body composition, using the Tanita BC-418MA body impedance analyser (BIA), based on transmitting an electrical current through the trunk, arms and legs [16]. Participants stand barefoot on the analyser's foot pads and grip the metal hand electrodes, while bio-impedance measurements were automatically taken and electronically transferred to the assessment centre’s IT system [17]. The device utilises an in-built proprietary algorithm to estimate BMR from BIA measures of fat-free mass, given the participant’s age, sex, weight and height [18]. BMR estimates were re-measured among 20,036 participants between 2012 and 2013, using a similar protocol.

### 2.3 Diabetes outcomes

New cases of diabetes that occurred after recruitment were considered as a proxi for ‘incident’, based on the first non-fatal or fatal event ever recorded in the Hospital Episode Statistics (HES) or the death certificate, during follow-up of the cohort. Diabetes events were classified using the International Classification of Diseases, 10th Revision (ICD-10) codes [19]. Diabetes subtypes (type 2, type 1 [included as a ‘negative control’], or unspecified) and specific complications that occurred among those who have had a diagnosis of diabetes after recruitment, were defined as the first event ever occurred among diabetes cases, specifically microvascular complications (retinopathy, neuropathy, peripheral vascular disease, or any other unspecified), macrovascular events (stroke, cardiac, and atherosclerotic diseases), and diabetic coma, ketoacidosis and glycaemic disturbances (see Table S1 for specific ICD-10 codes). Participants who did not experience an event were censored at the end of the study or loss to follow-up, whichever occurred first.

### 2.4 Statistical analyses

Participants with missing or extreme BMR estimates at baseline (4%) were excluded. Further, pre-existing cases of diabetes at recruitment (9%) were excluded from analyses, to minimize potential reverse causation (e.g., the effect of diabetes on BMR levels). After additionally excluding those with missing covariate data (20%), 341,790 participants remained eligible for analyses (Fig. S1).

All analyses were conducted separately in men and in women, given that the estimated BMR values did not overlap considerably between sexes. Sex-specific socio-demographic, lifestyle and biological characteristics of the study participants at baseline were examined across sex-specific quintiles of estimated BMR and presented as counts and proportions (%), means and standard deviations (SD), or medians and interquartile ranges (IQR), as appropriate. Variation of the baseline characteristics by levels of estimated BMR quintiles were tested using analysis of variance and *Χ^2^* tests, as appropriate.

The overall incidence rates of diabetes across quintiles of estimated BMR (using group means) were calculated from group-specific events and their corresponding person-years at risk. Cox proportional hazard models with age-at-risk as the underlying time scale were used to estimate hazard ratios (HRs) and 95% confidence intervals (CIs) given higher estimated BMR levels with subsequent risk for new cases of diabetes, including specific subtypes and complications. Analyses were adjusted for age-at-risk (5 year-groups), assessment centre (England, Wales, Scotland), ethnicity (Caucasians, non-Caucasians), Townsend deprivation index (quintiles), habitual physical activity (quintiles of metabolic equivalent of task [MET]), habitual processed meat intake (none, 1 to <3, 3 times/week), habitual alcohol consumption (none, 1 to <3, ≥3 times/week), and smoking status (never, ever). (Table S3 presents the a-priori assessment of the independent relevance of each confounder to diabetes). Further, effect modification by strata of these characteristics was also assessed.

First, the shape of the association between sex-specific quintiles of estimated baseline levels of BMR and subsequent diabetes risk were described by plotting the estimated HRs and their corresponding 95% CIs, against the baseline means of each BMR quintile, thus enabling comparisons of estimates across any two categories (other than the reference group) [20]. Further, overall HRs were calculated per each SD higher estimated BMR across the full ranges of the sex-specific distributions. Extended assessment of classic mediating risk factors for diabetes and its macro or microvascular complications to test biological assumptions of increased weight and metabolic disturbances due to BMR, included sequential account for glycated haemoglobin HbA1c, triglycerides, high-density lipoprotein cholesterol [HDL-c], Cystatin-C and C-reactive protein [CRP] (considered as one SD higher levels at baseline). The selection of these candidate mediators was based on the hypothesised metabolic links between positive energy balance and excess lipids and insulin dysregulation, and were empirically assessed for dose–response relationships trough linear graphs within the data (Supplemental Fig. S3) [3], [4]. Missing biochemistry data (18.2%) were imputed using multiple imputation, conditioned on variables predictive of missingness, to ensure that HR estimates remained aligned with those from the fully adjusted model and directly comparable with mediation results [21]. A semi-quantitative estimate of the mediating role of these biological factors (considered together) was evaluated by the percentage change in the HRs calculated as [22] [(HR_confounders_ – HR_confounders+mediators_*100) / (HR_confounders_-1)]), and the corresponding change in *Χ^2^*.

Sensitivity analyses for the main associations observed included assessment of effect modification (within baseline strata of confounders, above), additional account for physical activity (by imputation for 23.3% of the missing data), the correlate between BMR and body mass index (with account for both residual BMI in tertiles), and additional account for fasting time. Separately, overall hazard estimates at a threshold for BMR (>1654 kcal/day in men and >1219 kcal/day in women, respectively), were calculated (given the observed curvilinear shapes of associations). Highly-reproducible BMR measures over time did not warrant correction for regression dilution (MacMahon-Peto regression dilution ratios [23] between baseline and resurvey BMR measures were 0.94 in men and 0.93 in women, Fig. S11).

The proportional hazards assumption for the Cox model was assessed by examining the plots of the Schoenfeld residuals and performing component-wise and global Grambsch-Therneau tests [24]. The main model (above) was chosen after extensively investigating potential reverse causation bias, with additional analyses conducted by excluding other chronic diseases at baseline assessment (i.e., vascular, kidney, liver, obstructive pulmonary diseases and cancers), the first 5 years of follow-up, probable type I diabetes, pre-diabetes (excluding those with HbA1c ≥ 5.7% in the absence of a diagnose), and separately, un-diagnosed diabetes (by further excluding those with HbA1c ≥ 6.5% in addition to pre-existing history of baseline diabetes).

All statistical analyses were performed in Stata version 18 and R version 4.2.1.

## 3 Results

### 3.1 Baseline characteristics

**Fig. 1:**
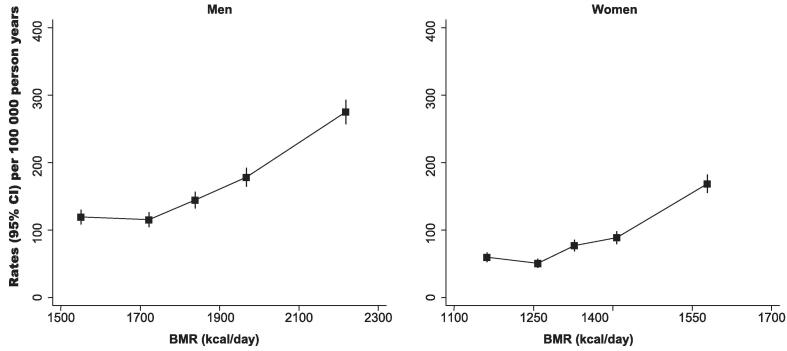
**Diabetes rates by levels of estimated BMR, stratified by sex** Diabetes rates per 100,000 person-years and corresponding 95% confidence intervals were derived from the group-specific number of events and their corresponding person-years at risk. BMR = Basal Metabolic Rate; CI = confidence intervals; kcal = kilocalories.

Among the 159,323 men and 182,467 women included in the main analyses the estimated mean (SD) age was 56 (8.1) years, and the mean estimated BMR was 1849 (234) kcal/day in men and 1340 (147) kcal/day in women (Table 1). Estimated mean BMR levels decreased with increase in age, and increased with increase in BMI, processed meat consumption, and triglyceride levels, but did not appear to vary substantially with physical activity time (Fig. S2). Women in the highest estimated BMR quintiles appeared more socioeconomically deprived, whereas men appeared somewhat more affluent. Participants in the highest estimated BMR quintile were more likely to consume processed meat frequently (≥3 times/week), be less physically active, and more likely to have ever smoked, compared to those in the lowest quintile (all p < 0.001) (Table S2**)**. Higher estimated BMR was associated with higher prevalence of diabetes (Fig. S4), and the estimated rate of new cases of diabetes increased steeply across the fifths of the BMR distributions from 115 to 257 cases/100,000 person-years in men, and from 57 to 147 cases in women, respectively (Fig. 1).

**Table 1:** Baseline characteristics among 341,790 participants from the UK Biobank.

**Characteristics**	**Men**	**Women**	**All**
**No. of participants**	159,323	182,467	341,790
**Sociodemographic factors**			
Age, years mean (SD)	56.2 (8.2)	55.7 (8.0)	55.9 (8.1)
Caucasian ethnicity*, n (%)	152,183 (95)	174,126 (95)	326,309 (95)
Townsend Deprivation Index, median (IQR)	−1.5 (3.0)	−1.5 (2.9)	−1.5 (3.0)
Assessment site in England, n (%)	141,060 (88)	161,297 (88)	302,357 (88)
**Lifestyle factors**			
Ever smoker, n (%)	77,540 (48)	73,217 (40)	150,757 (44)
Ever drinking, n (%)	151,223 (95)	168,316 (92)	319,539 (93)
Exercise MET, minutes/week, median (IQR)	105 (120)	100 (110)	100 (120)
Meat intake ≥ 3 times/week, n (%)	67,837 (43)	35,572 (19)	103,409 (30)
Vegetable intake, teaspoons/day, median (IQR)	4.0 (3)	5.0 (3)	4.0 (3)
Fruit intake, pieces/day, median (IQR)	2.0 (3)	3.0 (2)	3.0 (2)
**Anthropometry, mean (SD)**			
Basal metabolic rate, kcal/day, mean (SD)	1849 (234)	1340 (147)	1577 (319)
Height, cm	176 (7)	163 (6)	169 (9.2)
Weight, kg	85 (13)	70 (13)	77 (15)
Body mass index, kg/m^2^	27 (4)	27 (5)	27 (4)
**Biochemical measures**ϯ			
HbA1c %, mean (SD)	5.3 (0.4)	5.3 (0.3)	5.3 (0.4)
HbA1c ≥ 6.5% **, n (%)	9,836 (6)	12,181 (7)	22,017 (6)
HDL-c, mmol/L, mean (SD)	1.3 (0.3)	1.6 (0.4)	1.5 (0.4)
LDL-c, mmol/L, mean (SD)	3.6 (0.8)	3.6 (0.8)	3.6 (0.8)
Triglycerides, mmol/L, median (IQR)	1.7 (1.2)	1.3 (0.9)	1.4 (1.1)
Cystatin-C, mg/L, median (IQR)	0.9 (0.2)	0.8 (0.2)	0.9 (0.2)
C-reactive protein, median (IQR)	1.2 (1.7)	1.2 (2.0)	1.2 (1.9)

HbA1c = Glycated haemoglobin A1c; HDL-c=High-density lipoprotein cholesterol; IQR = Interquartile range; LDL-c = Low-density lipoprotein cholesterol; MET = Metabolic equivalent of task; SD=Standard deviation.

* Among men,1.8% were Asians, 1.2% Afro-Caribbeans, 0.5% Mixed and 1.0% of other ethnicity. Among women, 1.6% were Asians, 1.3% Afro-Caribbeans, 0.7% Mixed and 1.0% of other ethnicities.

ϯ Values among a subset of 279,579 participants with complete measures.

** Indicative of undiagnosed diabetes at baseline.

### 3.2 BMR and risk for diabetes and diabetic complications

During a mean follow-up of 12 years, 4,626 new diabetes cases (63.5% in men) were recorded at ages 35 to 75 years among those without known history at recruitment (with the earliest and latest cases being recorded at 7 years and 14 years after recruitment, respectively). The estimated diabetes incidence rate was 112 cases per 100,000 person-years, somewhat higher in men than in women (Fig. 1). Most of the accrued cases were from T2DM (n = 3,659, 65.2% in men), and among all the participants who had any type of diabetes post-recruitment, 36% developed specific complications (39% in men), but fatalities were low (53 deaths only).

**Fig. 2:**
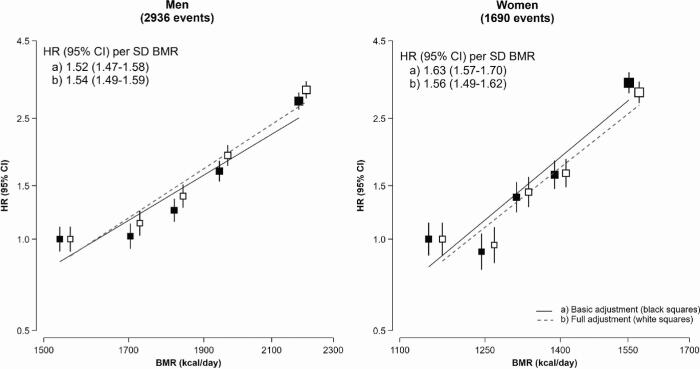
**Relevance of estimated BMR to diabetes risk, by sex** Hazard ratios were calculated from multivariable Cox models, and are shown as squares, plotted against the estimated mean BMR in each category, with the area of each square being inversely proportional to the variance of the log-HR and the corresponding vertical bars indicating the 95% confidence intervals. Black squares represent HR estimates after adjustment for age-at-risk (5 groups). White squares represent HR estimates after additional adjustment for ethnicity, Townsend Deprivation Index, processed meat intake, alcohol drinking, smoking status and assessment centre. Continuous estimates were calculated per each standard deviation higher estimated BMR (corresponding to 7.9 kcal/day in men and 9.1 kcal/day in women) across the full ranges. Residual-BMI adjusted HRs were 1.52 (1.46–1.57) in men and 1.54 (1.47–1.60) in women respectively (Supplemental Fig. S5). Regression dilution corrected HRs per each standard deviation higher usual BMR were 1.58 (95% CI 1.53–1.64) for men and 1.61 (1.54–1.68) for women. (Supplemental Fig. S11 shows the regression dilution ratios of 0.94 in mean and 0.93 in women). Exclusions as per Table1. BMR = Basal Metabolic Rate; CI = confidence intervals; HR = hazard ratios; kcal = kilocalories.

Overall, there was a positive association between higher estimated BMR levels and subsequent risk for diabetes, both in men as in women (Fig. 2). Compared to those within the bottom fifth of the estimated BMR levels at baseline, the HR for diabetes increased from 1.10 (95% CIs 1.00–1.21) to 3.17 (2.96–3.38) across the fifths of the distribution among men, and from 0.91 (0.79–1.04) to 3.13 (2.88–3.40) among women. Various medical or follow-up exclusions did not indicate reverse-association (Figs. S6-S8), and sensitivity analyses with additional exclusions of baseline HbA1c ≥ 5.7% indicative of pre-diabetes or un-diagnosed diabetes (Figs. S6**F and S8**), additional account for fasting time (Fig. S6**B**) or analyses imputed physical activity data supported the main findings **(**Table S4**)**. Furthermore, the association between estimated BMR and diabetes risk remained materially unchanged after mutual adjustment for residual BMI (representing BMI levels after its correlation with BMR was accounted for) (Fig. S5).

**Fig. 3:**
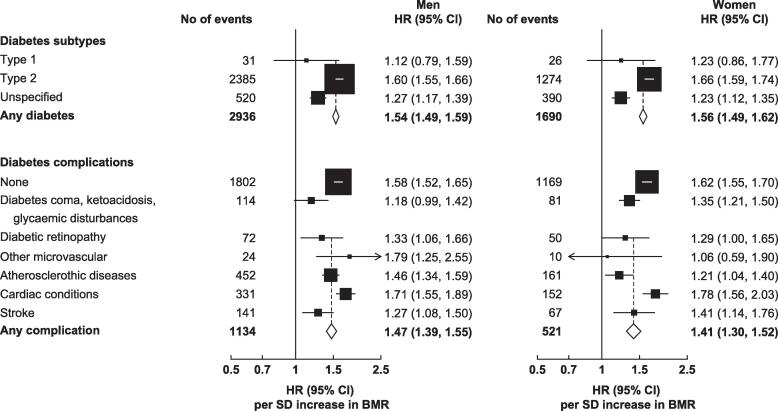
**Associations of estimated BMR with risk of diabetes subtypes and diabetes complications, by sex** Estimates and conventions as per fully adjusted model in Fig. 2, now with estimated BMR as a continuum per one standard deviation higher level. The diamond and the dashed vertical line represent the overall hazards. Of all diabetes events, only 53 were fatal. (Supplemental Table S1 shows the ICD-10 classification of the diabetes subtypes and of the specific complications that occurred among those with incident diabetes). BMR = Basal Metabolic Rate; CI = confidence intervals; HR = hazard ratios; kcal = kilocalories; SD = standard deviation.

Among men, every SD increase in estimated baseline BMR was associated with a 54% higher risk for diabetes (HR 1.54, 1.49–1.59), and a 56% higher risk in women (HR 1.56, 1.49–1.62) (independent of age-at-risk and all the confounders considered). The associations were essentially driven by the risk conferred to type 2 diabetes (HRs 1.60, 1.55–1.66 in men and 1.66, 1.59–1.74 in women), while for type 1 diabetes for which the low number of cases did not allow sufficient credibility, the associations appeared largely null (HRs 1.12, 0.79–1.59 in men and 1.23, 0.86–1.77 in women; Fig. 3).

The risk for diabetes complications with every SD higher estimated BMR among men was strong for diabetic retinopathy (HR 1.33, 1.06–1.66) and cardiac events (HR 1.71, 1.55–1.89), and more moderate for diabetic coma, ketoacidosis and glycaemic disturbances (HR 1.18, 0.99–1.42) or stroke events (HR 1.27; 1.08–1.50) (Fig. 3). In women, the risk for diabetes complications was broadly comparable to the risk observed in men for diabetic retinopathy (HR 1.29, 1.00–1.65) and cardiac complications (HR 1.78, 1.56–2.03), but much larger for diabetic coma, ketoacidosis and glycaemic disturbances (HR 1.35, 1.21–1.50), and stroke 1.41 (1.14–1.76). Furthermore, the risk observed for any other atherosclerotic disease complications was stronger in men than in women (HRs 1.46, 1.34–1.59 versus 1.21, 1.04–1.40) (Fig. 3). Estimates were marginally stronger by 5–10% when conservative threshold levels were applied for BMR (>1654 kcal/day in men and >1219 kcal/day in women). Specifically, the overall HRs for diabetes risk were 1.60 (1.54, 1.67) in men and 1.62 (1.54–1.70) in women, and the HRs for any diabetic complications were 1.57 (1.46–1.68) in men and 1.46 (1.33–1.60) in women, respectively (Fig. S9).

**Fig. 4:**
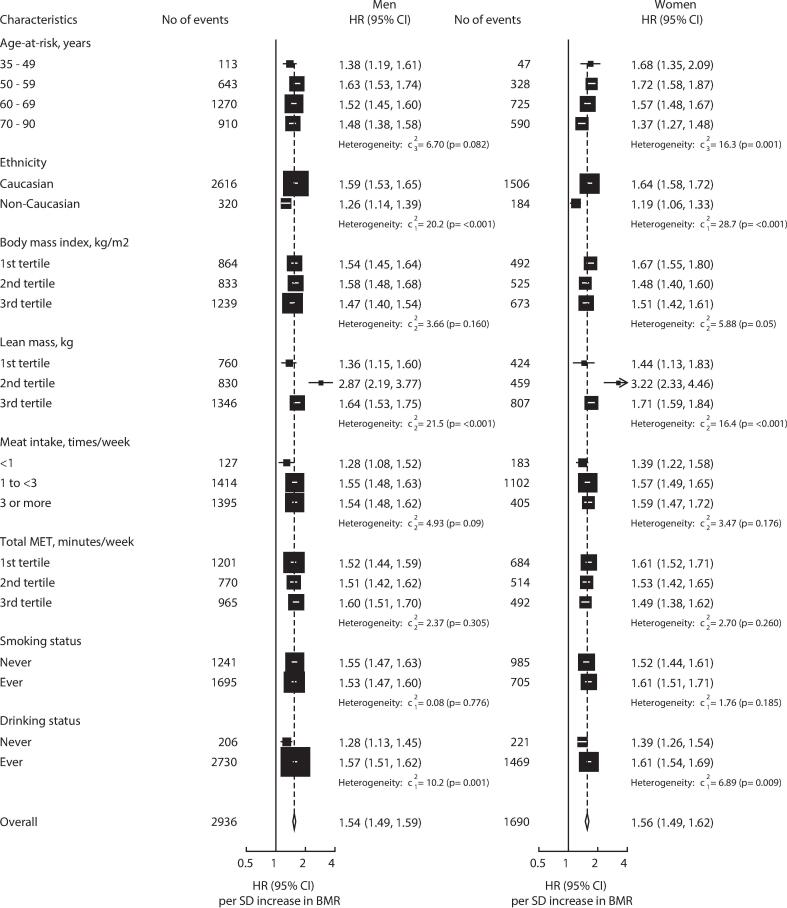
**Associations of estimated BMR with risk of diabetes, by sex and various characteristics at baseline** Analyses and conventions as per Fig. 3, now within strata of various characteristics as indicated in the plot. Tertiles of absolute residual BMI values were used to test for interaction (due to the high correlation between BMI and BMR). The number of diabetes cases among Asians was limited to only 138 cases in men and 85 cases in women, and among Afro-Caribbeans to only 105 cases in men and 46 cases in women. χ2 and p-values were estimated by likelihood-ratio tests of heterogeneity. BMR = Basal Metabolic Rate; BMI = body mass index; CI = confidence intervals; HR = hazard ratios; kcal = kilocalories; SD = standard deviation; χ2 = chi-squared statistic.

When the overall strength of the association between higher estimated BMR with new cases of diabetes was compared across various characteristics (Fig. 4), no evidence for effect modification was found for variation in BMI, frequency of processed meat consumption, weekly hours spent on physical activity, or smoking status (at the time BMR was measured). However, associations were stronger at younger than at older ages among women (HRs 1.68, 1.35–2.09 versus 1.37, 1.27–1.48; p for heterogeneity 0.001, p for trend 0.001) but not in men (HRs 1.38, 1.19–1.61 versus 1.48, 1.38–1.58, p for heterogeneity 0.082, p for trend 0.955). Moreover, associations were stronger for Caucasian than non-Caucasian participants (HRs 1.59, 1.53–1.65 versus 1.26, 1.14–1.39 in men, and 1.64, 1.58–1.72 versus.1.19,1.06–1.33 in women, p for heterogeneity <0.001), and about twice as strong among men who ever drink (p for heterogeneity  0.001) but not in women (p for heterogeneity  0.009).

### 3.3 Mediators of BMR associated risk for diabetes and its complications

**Fig. 5:**
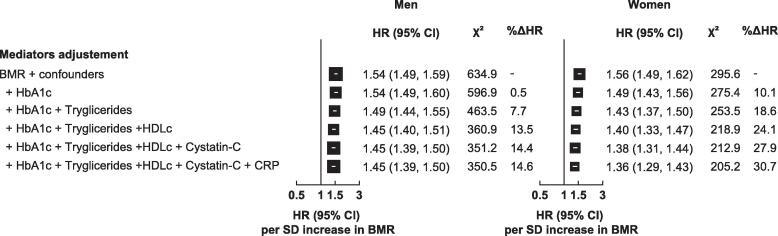
**Association of estimated BMR with risk of diabetes and its complications, given selected vascular-metabolic mediators, by sex** Analyses and conventions as per Fig. 3, now with each candidate mediator considered as increase per one standard deviation of measured levels at baseline (for comparison), and with additional account for assay batch and fasting time. A subset of 132,171 men and 147,408 women had complete biochemistry data, hence imputation for the remaining 62,220 (18.2%) participants included in the main analyses was performed. The shapes of the association between higher estimated BMR and each candidate mediator considered was first assessed with linear plots (e.g. dose–response associations) and regression models adjusted for age and sex (as shown in Supplemental Fig. S3). The percentage change in HR attributed to mediators was calculated to the 3rd decimal point as follows: [(HRconfounders-HRconfounders + mediators)*100)/(HRconfounders-1)]. BMR = Basal Metabolic Rate; CI = confidence intervals; CRP=C-reactive protein; HbA1c = Glycated haemoglobin 1c; HDL-c=High-density lipoprotein cholesterol; HR = hazard ratios; kcal = kilocalories; SD = standard deviation; χ2 = chi-squared statistic; %Δ = percent change.

Higher estimated BMR was associated with higher levels of BMI and triglycerides and lower levels of HDL-cholesterol, in a dose–response fashion, while the associations with glycated haemoglobin A1c, Cystatin-C and C-reactive protein were somewhat weaker, albeit linear throughout the ranges studied (Fig. S3), supporting a relationship between estimated BMR and vascular-metabolic disturbances. Sequential account for the candidate mediating biomarkers attenuated the overall association of estimated BMR with subsequent risk for diabetes from 1.54 (1.49–1.59) to 1.45 (1.39–1.50) in men, and from 1.56 (1.49–1.62) to 1.36 (1.29–1.43) in women (Fig. 5), largely independent of residual BMI (Fig. S10). Among women, these mediators combined explained almost one-third of the observed association between estimated BMR and diabetes risk (change in *χ*^2^ from 295.6 to 205.2), with HbA1c, triglycerides, HDL-cholesterol, Cystatin-C and CRP each explaining 10%, 9%, 6%, 4% and 3%, respectively. Among men, all mediators combined explained less than a fifth of the main association of estimated BMR with diabetes risk (change in *χ*^2^ from 635 to 350.5), with triglycerides being most relevant, and explaining 7% of the association.

## 4 Discussion

In this large prospective study of 341,790 UK adults, we found strong positive associations between higher BMR and subsequent risk for diabetes, irrespective of sex. Overall, higher estimated BMR was associated with a 55% higher risk of diabetes, largely driven by the risk with T2DM and major diabetic complications, such as cardiac events and diabetic coma, ketoacidosis and glycaemic disturbances. Albeit the risk observed was largely independent of the residual levels of BMI (namely, the residual BMI that was not correlated to BMR), circulating glycaemic, lipids, renal and inflammatory factors explained one-fifth to one-third of the association between estimated BMR and subsequent risk for diabetes, including its vascular and other complications.

Our main finding that higher estimated BMR is associated with a higher risk of diabetes contradicts two previous studies that reported null associations. One small prospective study of 233 participants who accrued two diabetic events over one year of follow-up, resting metabolic rate (i.e., the energy expended at rest in less stringent conditions compared to measurement conditions for BMR), null risk was reported for metabolic syndrome or diabetes (HR 1.00, 95% CI 0.996–1.005, *p* = 0.86) [25]. Although this study offers an advantage by measuring resting metabolic rate directly from calorimetry (e.g., the gold standard), the limited sample limits any reliability of the findings and any comparisons. One univariable two-sample MR using the UK Biobank data assessed the causal effect of BMR on T2DM and reported null associations [13]. Our findings are comparable to those from a more recent two-sample MR examining the causal effect of BMR on the risk of diabetes among participants from the DIAMATE consortium and the UK biobank, [12] which reported that every SD increase in genetically-determined BMR was associated with a 49% higher odds of T2DM (OR 1.49, 95% CI 1.31–1.70). By contrast to our findings suggesting insignificant attenuation of diabetes risk when BMR and BMI are considered together, this MR study suggested that mutual adjustment for genetically-determined BMI reverses the association of genetically-determined BMR with diabetes risk (OR 0.69, 0.18–2.40). As our analysis suggests, the association of estimated BMR with diabetes risk and diabetic complications described in this report is explained to a degree by a shared group of vascular-metabolic mediators, and in particular higher levels of triglycerides, which are commonly implicated in the pathology of excess adiposity to risk for diabetes, as well as vascular diabetic complications [26]. Our more novel findings, consist of the differences in risk between Caucasian and non-Caucasians, opposite to associations of BMI and diabetes which are stronger in non-Caucasian than in Caucasians, [27] albeit the limited number of Asian and African sub-populations represented in the UK biobank, together with the extremely low number of cases among these ethic groups, limited our capacity to further disentangle ethnic-specific differences. Furthermore, the discrete gender differences we observed for a higher risk for diabetic cardiac disease and diabetic coma, ketoacidosis and glycaemic disturbances among women, but a stronger risk for diabetic stroke among men, invite further assessment of the potential role of menopausal changes in the metabolic profiles and potential gender-differences in the management of diabetic therapies.

Although not fully understood, several mechanisms have been proposed to explain the positive association between BMR and diabetes. While BMR is traditionally considered as a marker of energy expenditure and metabolic health, accumulating evidence suggests that higher BMR may reflect upregulated metabolic activity and mitochondrial uncoupling at the cellular level, [28] which can lead to inefficient energy utilisation and impaired insulin sensitivity in peripheral tissues. Elevated BMR has also linked to disturbances in the sympathetic nervous system and hypothalamic–pituitary–adrenal axis, thereby promoting metabolic pathways that favour glucose overproduction, lipolysis, and chronic inflammation [29], [30]. Our findings suggesting the important mediating role of higher glycaemic and triglyceride levels, converges towards such potential metabolomic processes linking BMR with risk for diabetes, albeit evidence from clinical trials is warranted to establish the relevant interventions. Additionally, individuals with inherently elevated BMR may compensate for increased energy expenditure by consuming more calories, potentially leading to excess weight and fat accumulation, well-established risk factors for T2DM [4].

Our study also highlights strong associations between estimated BMR and risk for diabetic coma, ketoacidosis and glycaemic disturbances and major macrovascular complications in individuals with T2DM. The association between higher estimated BMR and the composite of myocardial infarction, heart failure, angina, and hypertensive heart disease in our study, may reflect previously hypothesised underlying autonomic imbalance, chronic sympathetic activation, or low-grade inflammation, as mechanisms underlying cardiac morbidity in diabetes [31], [32]. Furthermore, the positive association between estimated BMR and diabetic coma, ketoacidosis and glycaemic disturbances may reflect excess metabolic stress or autonomic dysregulation, known triggers of acute glycaemic decompensation [33], [34], [35]. In contrast, the moderate association observed with atherosclerotic complications in men only in our study, might suggests sex-specific susceptibility to chronic vascular injury under metabolic strain. Men with type 2 diabetes tend to exhibit greater visceral adiposity and more adverse lipid profiles than women, potentially contributing to an elevated atherogenic burden [36]. However, given most women in the cohort were likely postmenopausal (mean age 56 years), a stage of increased cardiometabolic risk due to declining oestrogen, [37] the lack of a clear association with atherosclerotic complications in women in our study may reflect limited statistical power, residual hormonal protection, or divergent metabolic pathways [38]. Further large-scale longitudinal and molecular studies are needed to clarify the role of BMR in risk for diverse diabetes complications. Indeed, our mediation analysis showed that established cardiometabolic risk factors partially explained the link between higher estimated BMR and diabetes and its major vascular complications, with HbA1c, triglycerides, HDLc and Cystatin-C explaining ∼ 18% of the risk in men and ∼ 33% of the risk in women. This difference suggests that, in women, elevated estimated BMR may exert a greater influence through metabolic dysregulation, consequent excess weight, and targeting these risk factors could mitigate some of the BMR-associated risk of diabetes. Individual-participant studies incorporating polygenic risk scores, gene–environment interaction models, or genotype-stratified analyses are warranted to disentangle the biological pathways linking BMR to diabetes risk.

To our knowledge, this is the first largest prospective analysis to comprehensively examine the relationship between estimated BMR and diabetes in various scenarios. It is the first to include diabetes complications, employing consistent methods to address confounding, reverse causality, effect modification, and to evaluate sex-differences including mediation of classic vascular-metabolic risk factors. Moreover, by employing a triangulation approach to delineate the influence of residual BMI on BMR across varying assumptions, this study provides consistent findings across different scenarios, highlights the novel finding that it is high, not low estimated BMR that increases risk for diabetes, advancing the current discourse on the BMI-BMR relevance to diabetes risk.

Nonetheless, several limitations merit consideration. First, BMR was estimated using bioimpedance analysis rather than calorimetry-based assessment, which although not the gold standard it relates reliably (r = 0.90) with indirect calorimetry measures, but might still over-estimates the true associations [18]. Moreover, diabetic retinopathy, stroke events and other microvascular complications were less powered, hence we cannot fully account for the play of chance [38]. Additionally, although our study population are largely comparable to the wider UK Biobank (Table S5), the UKB cohort are healthier and socioeconomically more advantaged, compared to the entire UK population. Despite this, relative risk estimates from non-representative cohorts can provide valuable insights into exposure-disease associations [39]. Lastly, any association we report cannot fully address residual confounding from unmeasured factors, notably habits that may influence the BMR estimates at the time of the measurement (e.g. exercise performed or dietary and alcohol consumption on the day of measurement), or genetic liability, given that BMR’s estimated heritability is around 40% [40] and ethnicity appeared to importantly modify the observed hazards in our study**.** Consequently, causality cannot be established from this observational design.

## 5 Conclusion

Our findings support the possibility that elevated estimated BMR is positively associated with a higher risk for subsequent diabetes and its complications, both in men as in women, and largely independent of residual BMI. Up to 30% of the risk of BMR with diabetes and its vascular-metabolic complications appeared mediated by glucose, triglycerides, high-density cholesterol and inflammatory markers, suggesting that controlling for these factors, may mitigate the risk due to BMR, particularly among women. Nonetheless, understanding the underlying mechanisms involved merit further investigation.

## CRediT authorship contribution statement

**Joseph Frimpong:** Writing – original draft, Visualization, Investigation, Formal analysis, Data curation. **Andrew Browne:** Writing – review & editing, Validation, Supervision, Methodology, Formal analysis. **Wrivu Martin:** Writing – review & editing. **Pallavi Kaushik:** Writing – review & editing, Software, Resources, Data curation. **Louisa Gnatiuc Friedrichs:** Writing – review & editing, Validation, Supervision, Resources, Project administration, Methodology, Funding acquisition, Conceptualization.

## Declaration of competing interest

The authors declare that they have no known competing financial interests or personal relationships that could have appeared to influence the work reported in this paper.
