# Supplementary material for: Basal metabolic rate and risk for diabetes and its complications among 341,790 adults from the UK Biobank
Source: Diabetes Res Clin Pract. 2026 May;235:113198. doi: 10.1016/j.diabres.2026.113198 (PMC13328060; doi:10.1016/j.diabres.2026.113198)
Supplement: Supplementary Data 1 [file mmc1.pdf]

# Basal metabolic rate and risk for diabetes and its complications among 341,790 adults from the UK Biobank

## Supplementary tables

Table S1: ICD-10 classification of diabetes subtypes and diabetic complications ..... 2

Table S2: Baseline characteristics by levels of estimated BMR (fifths) and by sex….3

Table S3: Minimally adjusted associations of individual covariates with risk from diabetes among the UK Biobank participants........................................................ 4

Table S4: Sensitivity analyses of main model, now with imputed data and additional adjustment for physical activity among 194,643 men and 244,948 women .............. 5

Table S5: Baseline characteristics among participants included in the main analyses versus the full cohort (without any exclusions), stratified by sex ............................... 6

## Supplementary figures

Figure S1: Participant selection diagram for the analysis of basal metabolic rate and diabetes in the UK Biobank ........................................................................... 7

Figure S2: Univariable associations of estimated BMR with selected characteristics at baseline, by sex.......................................................................................... 8

Figure S3: Estimated BMR versus selected mediators at baseline, by sex................ 9

Figure S4: Prevalence of diabetes by levels of estimated BMR A-B) before recruitment; and C-D) at recruitment, stratified by sex .......................................... 10

Figure S5: Relevance of estimated BMR to diabetes, with additional adjustment for residual BMI ............................................................................................. 11

## Sensitivity Analyses

Figure S6: The association between estimated estimated BMR and diabetes, with different exclusions and adjustements

A) No medical exclusions ......................................................................... 12

B) Excluding pre-existing diabetes at baseline and account for fasting time............ 12

C) Excluding pre-existing diabetes and other chronic disease at baseline…………13

D) Excluding any pre-existing chronic disease at baseline and the first 5 years of follow-up …………………………………………………………………………..…13

E) Excluding probably Type I diabetes from the main composite outcome …………14

F) Excluding those with HbA1c ≥ 5.7% in addition to excluding diabetes …..………14

Figure S7: Associations of estimated BMR with diabetes subtypes and complications by sex, after further excluding other prior chronic diseases at baseline .................. 15

Figure S8: Association of estimated BMR and diabetes subtypes and complications by sex, after further excluding participants with HbA1c≥5.7% among those undiagnosed with diabetes at baseline................................................................. 16

Figure S9: Associations of estimated BMR with diabetes subtypes and complications above BMR cut-off >1654 kcal/day in men and >1219 kcal/day in women .............. 17

Figure S10: Associations of estimated BMR with diabetes, given candidate vascular-metabolic mediators by sex, after additional account for residual BMI ……….…..…18

Figure S11: Regression to the mean of estimated BMR measures between baseline and resurvey, by sex ................................................................................ 19

**Table S1: ICD-10 classification of diabetes and its complications**

| Description | ICD-10 codes |
|---|---|
| **Any diabetic events*** | Any of the below |
| **Diabetes subtypes** | |
| Type 1 | E10.0-E10.9 |
| Type 2 | E11.0-E11.9 |
| Unspecified | E12.0-E16.2, G59.0, G63.2, G73.0, G99.0, H28.0, H36.0, I79.2, I79.8, M14.2, M14.6, N08.3, O24.0-4, O24.9 |
| **Any diabetic complications**** | Any of the below |
| Diabetes coma, ketoacidosis, glycaemic disturbances | E10.0-1, E11.0-1, E12.0-1, E13.0-1, E14.0-1, E15, E16.0-2 |
| **Diabetes with microvascular complications** | Any of the below |
| Peripheral vascular disease | I79.2, E10.5, E11.5, E12.5, E13.5, E14.5 |
| Renal disease | N08.3, E10.2, E11.2, E12.2, E13.2, E14.2 |
| Neurological disease | G73.0, G99.0, G59.0, G63.2, E10.4, E11.4, E12.4, E13.4, E14.4 |
| Ophthalmic disease | H28.0, H36.0, E10.3, E11.3, E12.3, E13.3, E14.3 |
| Any other/unspecified | M14.2, M14.6, E10.7-8, E11.7-8, E12.7-8, E13.7-8, E14.7-8, I79.8 |
| **Diabetes with macrovascular complications** | Any of the below |
| Cardiac (hypertensive cardiac disease, angina, myocardial infarction, heart failure) | I11.0, I11.9, I20.0-8, - I21.9, I24.0, I24.8-9, I50.0-.9 |
| Stroke (incl. transient ischaemic attack) | I61*, I63.0-9, I64.0, G45.0-9 |
| Atherosclerothic disease (other than above) | I25.0-1, I65.0- I70.9 |

*Non-fatal or fatal events were defined as the first mention in any part of the HES records or the death certificate.
**Considered only if they occurred among those with diabetes.
Reference https://icd.who.int/browse10/2019/en#/E10-E14

Table S2: Baseline characteristics by levels of estimated basal metabolic rate among 159,323 men and 182,467 women from the UK Biobank

| | Basal Metabolic Rate (fifths), kcal/day | | | | | | | | | |
|---|---|---|---|---|---|---|---|---|---|---|
| Characteristic | Men | | | | | Women | | | | |
| | Q1 | Q2 | Q3 | Q4 | Q5 | Q1 | Q2 | Q3 | Q4 | Q5 |
| **No. of participants** | 32,042 | 31,879 | 31,844 | 31,844 | 31,714 | 36,882 | 36,622 | 36,312 | 36,410 | 36,241 |
| **Sociodemographic factors** | | | | | | | | | | |
| Age years, mean (SD) | 58.6 (7.8) | 57.3 (8.0) | 56.3 (8.2) | 55.2 (8.2) | 53.5 (8.1) | 57.9 (7.6) | 56.4 (7.9) | 55.6 (8.0) | 54.8 (8.0) | 53.7 (7.9) |
| Ethnicity, n (%) | | | | | | | | | | |
| Caucasian | 29,566 (92) | 30,441 (95) | 30,662 (96) | 30,794 (97) | 30,720 (97%) | 34,430 (93) | 35,159 (96) | 35,028 (96) | 35,063 (96) | 34,446 (95) |
| Non-Caucasians* | 2,476 (8) | 1,438 (4) | 1,182 (4) | 1,050 (3) | 994 (3) | 2,452 (7) | 1,463 (4) | 1,284 (3) | 1,347 (4) | 1,795 (5) |
| Townsend Index, median (IQR) | -1.2 (3.2) | -1.6 (3.0) | -1.7 (2.9) | -1.7 (2.9) | -1.5 (3.0) | -1.6 (2.9) | -1.7 (2.8) | -1.6 (2.9) | -1.5 (2.9) | -1.2 (3.1) |
| Assessment site, n (%) | | | | | | | | | | |
| England | 28,036 (87) | 28,079 (88) | 28,294 (89) | 28,337 (89) | 28,314 (89) | 32,355 (88) | 32,244 (88) | 32,089 (88) | 32,256 (89) | 32,353 (89) |
| Scotland | 2,656 (8) | 2,459 (8) | 2,257 (7) | 2,209 (7) | 2,023 (6) | 3,124 (8) | 2,897 (8) | 2,754 (8) | 2,595 (7) | 2,386 (7) |
| Wales | 1,350 (4) | 1,341 (4) | 1,293 (4) | 1,298 (4) | 1,377 (4) | 1,403 (4) | 1,481 (4) | 1,469 (4) | 1,559 (4) | 1,502 (4) |
| **Lifestyle factors** | | | | | | | | | | |
| Ever smoker, n (%) | 15,372 (48) | 15,214 (48) | 15,357 (48) | 15,735 (49) | 15,862 (50) | 13,773 (37) | 14,448 (39) | 14,549 (40) | 15,011 (41) | 15,436 (43) |
| Ever alcohol intake, n (%) | 30,554 (93) | 31,187 (95) | 30,898 (96) | 30,410 (96) | 28,174 (96) | 34,134 (90) | 34,753 (93) | 34,922 (93) | 33,599 (93) | 30,908 (92) |
| Exercise MET minutes/week, median (IQR) | 105 (120) | 105 (120) | 105 (120) | 105 (120) | 100.0 (128) | 105 (120) | 105 (120) | 100 (115) | 100 (105) | 90 (100) |
| Meat intake ≥3 times/week, n (%) | 12,810 (40) | 12,970 (41) | 13,287 (42) | 13,788 (43) | 14,982 (47) | 6,200 (17) | 6,510 (18) | 6,853 (19) | 7,417 (20) | 8,592 (24) |
| Vegetable intake, teaspns/day, median (IQR) | 4.0 (3) | 4.0 (3) | 4.0 (3) | 4.0 (3) | 4.0 (3) | 4.0 (3) | 4.5 (3) | 4.5 (3) | 5.0 (3) | 5.0 (3) |
| Fruit intake, pieces/day, median (IQR) | 2.0 (3) | 2.0 (3) | 2.0 (3) | 2.0 (3) | 2.0 (3) | 3.0 (3) | 3.0 (2) | 3.0 (2) | 3.0 (2) | 3.0 (2) |
| **BMR and Anthropometry** | | | | | | | | | | |
| Basal metabolic rate, mean (SD) | 1548 (88) | 1717 (34) | 1831 (33) | 1956 (42) | 2198 (144) | 1161 (47) | 1256 (20) | 1323 (20) | 1400 (27) | 1564 (102) |
| BMR group-ranges | <1654 | 1654 - 1773 | 1774 - 1887 | 1888 - 2034 | ≥2035 | <1219 | 1219 - 1288 | 1289 - 1356 | 1357 - 1449 | ≥1450 |
| Height, cm | 170 (6) | 174 (5) | 176 (5) | 178 (5) | 182 (6) | 158 (5) | 161 (5) | 163 (5) | 165 (7) | 167 (6) |
| Weight, kg | 70 (6) | 78 (5) | 84 (5) | 90 (6) | 102.9 (11) | 57 (5) | 63 (5) | 68 (5) | 74 (6) | 88 (11) |
| Body mass index, kg/m² | 24 (3) | 26 (3) | 27 (3) | 28 (3) | 31 (4) | 23 (23) | 24 (3) | 26 (3) | 28 (3) | 32 (5) |
| Fat mass, kg | 16 (5) | 19 (5) | 21 (6) | 23 (6) | 29 (8) | 18 (5) | 22 (5) | 24 (6) | 28 (6) | 37 (9) |
| Lean mass, kg | 54 (3) | 59 (1) | 63 (1) | 67 (2) | 74 (4) | 39 (2) | 42 (1) | 44 (1) | 46 (1) | 51 (3) |
| **Biochemical measures** | | | | | | | | | | |
| HbA1c, %, mean (SD) † | 5.3 (0.4) | 5.3 (0.4) | 5.3 (0.4) | 5.3 (0.5) | 5.4 (0.4) | 5.3 (0.3) | 5.3 (0.3) | 5.3 (0.4) | 5.3 (0.3) | 5.4 (0.4) |
| HbA1c ≥ 6.5%**, n (%) | 1,940 (6.1) | 1,878 (5.9) | 1,983 (6.2) | 1,989 (6.2) | 2,046 (6.5) | 2,370 (6.4%) | 2,393 (6.5) | 2,343 (6.5) | 2,431 (6.7) | 2,644 (7.3) |
| HDL-cholesterol, mmol/L, mean (SD) $ | 1.4 (0.3) | 1.3 (0.3) | 1.3 (0.3) | 1.3 (0.3) | 1.2 (0.3) | 1.7 (0.4) | 1.7 (0.4) | 1.6 (0.4) | 1.6 (0.4) | 1.5 (0.3) |
| LDL-cholesterol, mmol/L, mean (SD) †† | 3.5 (0.8) | 3.6 (0.8) | 3.6 (0.8) | 3.6 (0.8) | 3.6 (0.8) | 3.6 (0.8) | 3.6 (0.9) | 3.6 (0.9) | 3.6 (0.9) | 3.7 (0.8) |
| Triglycerides, mmol/L, median (IQR) ¥ | 1.4 (1.0) | 1.6 (1.1) | 1.6 (1.2) | 1.7 (1.3) | 1.9 (1.4) | 1.2 (0.7) | 1.2 (0.8) | 1.2 (0.8) | 1.3 (0.9) | 1.5 (1.0) |
| Cystatin-C, mg/L, median (IQR) £ | 0.9 (0.2) | 0.9 (0.2) | 0.9 (0.2) | 0.9 (0.2) | 0.9 (0.2) | 0.8 (0.2) | 0.8 (0.2) | 0.8 (0.2) | 0.8 (0.2) | 0.9 (0.2) |
| C-reactive protein | | | | | | | | | | |

Exclusions and conventions as per Table1. All p-values were <0.001, both in men as in women.

**Table S3: Minimally adjusted associations of individual covariates with risk from incident diabetes among the UK Biobank participants**

| | No Events / Total | HR (95% CIs) | Statistical test |
|---|---|---|---|
| **No. of participants** | 4,626 / 34,1790 | - | - |
| Assessment site | | | |
| Wales | 201 / 14,073 | Reference | ga (df) = 2, ga (p) < 0.001 |
| Scotland | 167 / 25,360 | 0.20 (0.17-0.25) | nl (df) = 1, nl (p) < 0.001 |
| England | 4,258 / 302,357 | 0.57 (0.49-0.65) | |
| Ethnicity | | | |
| Caucasians | 4,122 / 326,309 | Reference | ga (df) =1, ga (p) < 0.001 |
| Non-Caucasians | 504 / 15,481 | 4.33 (3.94-4.75) | |
| Townsend Deprivation Index, fifths | | | |
| Q1 (<-4.0) | 771 / 68,469 | Reference | ga (df) = 4, ga (p) < 0.001 |
| Q2 (-4.0 to <-2.9) | 848 / 68,304 | 1.12 (1.02-1.23) | nl (df) = 3, nl (p) < 0.001 |
| Q3 (-2.9 to <-1.5) | 891 / 68,639 | 1.21 (1.10-1.33) | |
| Q4 (-1.5 to <0.9) | 908 /68,114 | 1.35 (1.23-1.48) | |
| Q5 (≥0.9) | 1,208 / 68,264 | 1.89 (1.73-2.06) | |
| BMI, kg/m², fifths | | | |
| Q1 (<23.3) | 302 / 68,940 | Reference | ga (df) = 4, ga (p) < 0.001 |
| Q2 (23.3 to <25.4) | 490 / 70,357 | 1.32 (1.14-1.52) | nl (df) = 3, nl (p) < 0.001 |
| Q3 (25.4 to <27.4) | 740 / 68,870 | 1.85 (1.62-2.11) | |
| Q4 (27.4 to <30) | 1,068 / 65,512 | 2.78 (2.45-3.16) | |
| Q5 (≥30) | 2,026 / 68,111 | 5.68 (5.03-6.42) | |
| Physical activity (MET), hours/week, fifths | | | |
| Q1 (<50) | 1,350 / 77,869 | Reference | ga (df) = 4, ga (p) < 0.001 |
| Q2 (50 to <80) | 759 / 59,264 | 0.74 (0.67-0.80) | nl (df) =3, nl (p) < 0.001 |
| Q3 (80 to <120) | 820 / 69,411 | 0.68 (0.63-0.74) | |
| Q4 (120 to <200) | 806 / 69,236 | 0.63 (0.58-0.69) | |
| Q5 (≥ 200) | 891 / 66,010 | 0.67 (0.63-0.73) | |
| Processed meat, times/week | | | |
| None | 310 / 32,932 | Reference | ga (df) = 2, ga (p) <0.001 |
| 1 to <3 | 2,516 / 205,449 | 1.08 (0.96-1.22) | nl (df) =1, nl (p) < 0.001 |
| ≥3 | 1,800 / 103,409 | 1.42 (1.25-1.60) | |
| Alcohol intake, times/week | | | |
| Never | 427 / 22,251 | Reference | ga (df) = 2, ga (p) < 0.001 |
| <3 | 2,289 / 159,203 | 0.82 (0.74-0.91) | nl (df) = 1, nl (p) = 0.005 |
| ≥3 | 1,910 / 160,336 | 0.56 (0.50-0.62) | |
| Smoking | | | |
| Never | 2,226 / 191,033 | Reference | ga (df) = 1, ga (p) < 0.001 |
| Ever | 2,400 (51.9%) | 1.11 (1.04-1.18) | |
| Vegetables, teaspoons/day, fifths | | | |
| Q1 (<3) | 1,660 / 112,455 | Reference | ga (df) = 4, ga (p) < 0.001 |
| Q2 (3 to <4) | 854 / 69,712 | 0.78 (0.77-0.85) | nl (df) = 3, nl (p) < 0.001 |
| Q3 (4 to <5) | 617 / 49,314 | 0.78 (0.71-0.86) | |
| Q4 (5 to <6.5) | 541 / 40,113 | 0.86 (0.78-0.95) | |
| Q5 (≥6.5) | 905 / 67,874 | 0.85 (0.79-0.93) | |
| Fruits, pieces/day, fifths | | | |
| Q1 (<1) | 1,384 / 82,968 | Reference | ga (df) = 4, ga (p) <0.001 |
| Q2 (1 to <2) | 1,057 / 75,124 | 0.82 (0.76-0.89) | nl (df) = 3, nl (p) = 0.006 |
| Q3 (2 to <3) | 775 / 66,862 | 0.68 (0.62-0.74) | |
| Q4 (3 to <5) | 874 / 73,079 | 0.67 (0.62-0.74) | |
| Q5 (≥5) | 501 / 42,006 | 0.63 (0.56-0.69) | |

Global association (ga) and non-linearity (nl) of individual variables with diabetes were tested using Chi-square and Likelihood ratio tests respectively. Univariable HRs are adjusted for age at risk and sex only.
P-value <0.05 considered statistically significant.
BMI=Body mass index; MET=Metabolic equivalent of task; HR=Hazard ratio; CI=Confidence interval.

**Table S4: Sensitivity analyses of main model now with imputed data and additional adjustment for physical activity among 194,643 men and 244,948 women**

| | Main model | | Including imputed data | |
|---|---|---|---|---|
| **Baseline BMR** | **No. of events** | **HR (95% CI)** | **No. of events** | **HR (95% CI)** |
| **Men, BMR fifths** | | | | |
| Q1 | 458 | Reference (1.00) | 584 | Reference (1.00) |
| Q2 | 428 | 1.10 (0.96-1.26) | 583 | 1.19 (1.05-1.33) |
| Q3 | 518 | 1.44 (1.27-1.64) | 649 | 1.42 (1.27-1.59) |
| Q4 | 623 | 1.89 (1.67-2.14) | 787 | 1.89 (1.70-2.11) |
| Q5 | 909 | 3.17 (2.82-3.56) | 1153 | 3.21 (2.89-3.56) |
| Overall* | 2936 | 1.54 (1.49-1.59) | 3756 | 1.55 (1.50-1.60) |
| **Women, BMR fifths** | | | | |
| Q1 | 260 | Reference (1.00) | 388 | Reference (1.00) |
| Q2 | 204 | 0.91 (0.76-1.09) | 322 | 0.96 (0.83-1.11) |
| Q3 | 301 | 1.40 (1.19-1.66) | 431 | 1.36 (1.18-1.57) |
| Q4 | 328 | 1.61 (1.37-1.90) | 502 | 1.66 (1.45-1.90) |
| Q5 | 597 | 3.12 (2.69-3.63) | 874 | 3.13 (2.77-3.54) |
| Overall | 1690 | 1.56 (1.49-1.62) | 2517 | 1.56 (1.51-1.62) |

Estimates are in reference to the lower fifth (Q1), and confidence intervals (CIs) do not represent group-specific variances. Missing baseline physical activity data (22.2%) in the analysable sample (see Figure S1 for more details) were imputed using multiple imputation. Under the assumption that data was missing at random, all covariates that were predictive of missingness were included (baseline age, assessment centre, ethnicity, Townsend Deprivation Index, physical activity, processed meat intake, alcohol drinking, and smoking status).
*Overall estimates refer to HRs calculated per one standard deviation higher BMR. BMR=basal metabolic rate; HR=hazard ratios; CI=confidence interval.

**Table S5: Comparison of baseline characteristics among participants included in the main analyses versus the full cohort (without any exclusions), by sex**

| | Cohort For Main Analysis | | | Full Cohort | | |
|---|---|---|---|---|---|---|
| **Characteristics** | **Men** | **Women** | **p-value** | **Men** | **Women** | **p-value** |
| No. of participants | 159,323 | 182,467 | | 228,973 | 273,156 | |
| Person-years of follow up, mean (SD) | 12.0 (0.9) | 12.0 (0.9) | 0.072 | 12.1 (1.0) | 12.1 (1.0) | 0.067 |
| **Sociodemographic factors** | | | | | | |
| Age, years, mean (SD) | 56.2 (8.2) | 55.7 (8.0) | <0.001 | 56.7 (8.2) | 56.4 (8.0) | <0.001 |
| Ethnicity, n (%) | | | | | | |
| Caucasians | 152,183 (95.5) | 174,126 (95.4) | 0.208 | 215,121 (94.1) | 257,240 (94.3) | 0.003 |
| Non-Caucasians | 7,140 (4.5) | 8,341 (4.6) | | 13,406 (5.9) | 15,464 (5.7) | |
| Townsend Deprivation Index, median (IQR) | -1.5 (3.0) | -1.5 (2.9) | 0.489 | -1.2 (3.2) | -1.3 (3.0) | <0.001 |
| Assessment site, n (%) | | | | | | |
| England | 141,060 (88.5) | 161,297 (88.4) | 0.005 | 203,566 (88.9) | 241,930 (88.6) | <0.001 |
| Scotland | 11,604 (7.3) | 13,756 (7.5) | | 15,891 (6.9) | 19,939 (7.3) | |
| Wales | 6,659 (4.2) | 7,414 (4.1) | | 9,516 (4.2) | 11,287 (4.1) | |
| **Lifestyle factors** | | | | | | |
| Ever smoker, n (%) | 77,540 (48.7) | 73,217 (40.1) | <0.001 | 116,136 (51.0) | 109,721 (40.4) | <0.001 |
| Ever alcohol intake, n (%) | 151,223 (94.9) | 168,316 (92.2) | <0.001 | 169,124 (94.3) | 178,961 (91.6) | |
| Exercise MET, minutes/week, median (IQR) | 105.0 (120.0) | 100.0 (110.0) | <0.001 | 100.0 (125.0) | 100.0 (115.0) | <0.001 |
| Meat intake ≥3 times/week, n (%) | 67,837 (42.6) | 35,572 (19.5) | <0.001 | 99,318 (43.4) | 56,592 (20.7) | <0.001 |
| Vegetable intake, teaspoons/day, median (IQR) | 4.0 (3.0) | 5.0 (3.0) | <0.001 | 4.0 (3.0) | 4.5 (3.0) | <0.001 |
| Fruit intake, pieces/day, median (IQR) | 2.0 (3.0) | 3.0 (2.0) | <0.001 | 2.0 (3.0) | 3.0 (2.0) | <0.001 |
| **Anthropometry, mean (SD)** | | | | | | |
| Basal metabolic rate, kcal/day, mean (SD) | 1849.2 (234.1) | 1340.0 (146.7) | <0.001 | 1858.1 (248.8) | 1350.1 (161.2) | <0.001 |
| Height, cm | 176.0 (6.7) | 162.8 (6.2) | <0.001 | 175.6 (6.8) | 162.4 (6.3) | <0.001 |
| Weight, kg | 84.8 (13.1) | 70.1 (12.7) | <0.001 | 85.9 (14.4) | 71.5 (14.1) | <0.001 |
| Body mass index, kg/m² | 27.4 (3.8) | 26.5 (4.7) | <0.001 | 27.8 (4.2) | 27.1 (5.2) | <0.001 |
| Fat mass, kg | 21.4 (7.5) | 25.9 (9.2) | <0.001 | 22.3 (8.2) | 27.0 (10.1) | <0.001 |
| Lean mass, kg | 63.5 (7.4) | 44.3 (4.6) | <0.001 | 63.6 (7.8) | 44.5 (5.0) | <0.001 |
| **Biochemical measures** | | | | | | |
| HbA1c, %, mean (SD) | 5.3 (0.4) | 5.3 (0.3) | 0.008 | 5.5 (0.7) | 5.4 (0.5) | <0.001 |
| HbA1C ≥6.5%, n (%) | 9,836 (6.2) | 12,181 (6.7) | <0.001 | 26,801 (11.7) | 27,519 (10.1) | <0.001 |
| HDL-cholesterol, mmol/L, mean (SD) | 1.3 (0.3) | 1.6 (0.4) | <0.001 | 1.3 (0.3) | 1.6 (0.4) | <0.001 |
| LDL-cholesterol, mmol/L, mean (SD) | 3.6 (0.8) | 3.6 (0.8) | <0.001 | 3.5 (0.9) | 3.6 (0.9) | <0.001 |
| Triglycerides, mmol/L, median (IQR) | 1.7 (1.2) | 1.3 (0.9) | <0.001 | 1.7 (1.3) | 1.3 (0.9) | <0.001 |
| Cystatin-C, mg/L, median (IQR) | 0.9 (0.2) | 0.8 (0.2) | <0.001 | 0.9 (0.2) | 0.9 (0.2) | <0.001 |
| C-reactive protein, mg/L, median (IQR) | | | | | | |

Conventions as per Tables 1 and S2.

**Figure S1: Participant selection diagram for the analysis of basal metabolic rate and diabetes in the UK Biobank**

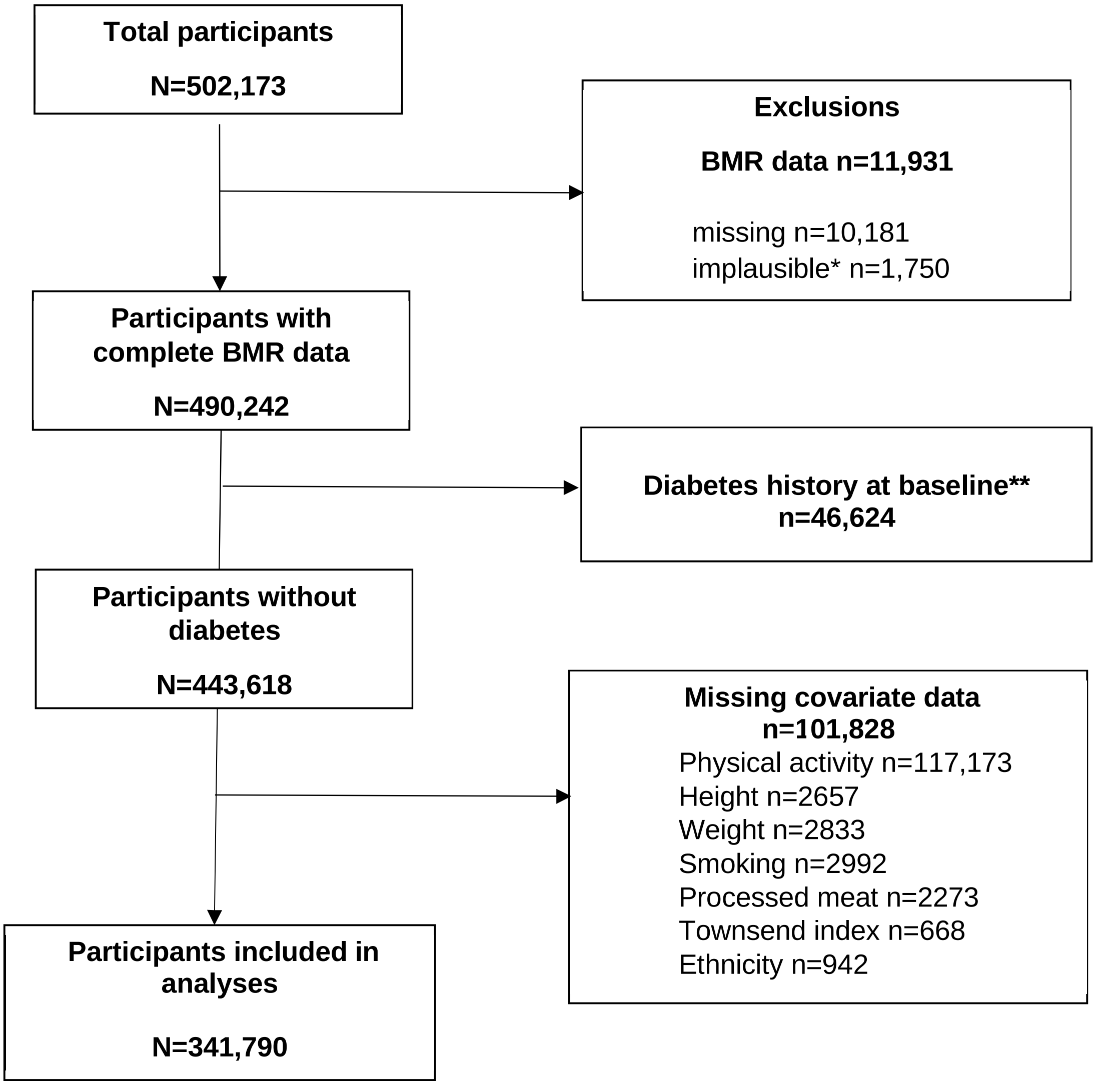


***** Implausible BMR values were defined as those exceeding ±4 standard deviations from the mean.

** Includes prior diabetes cases documented from Hospital Episode Statistics, classified by ICD-10 (see Table S1 for more details) and participant self-reported diabetes history at baseline assessment.

**Figure S2: Univariable associations of estimated BMR with selected characteristics at baseline, by sex**

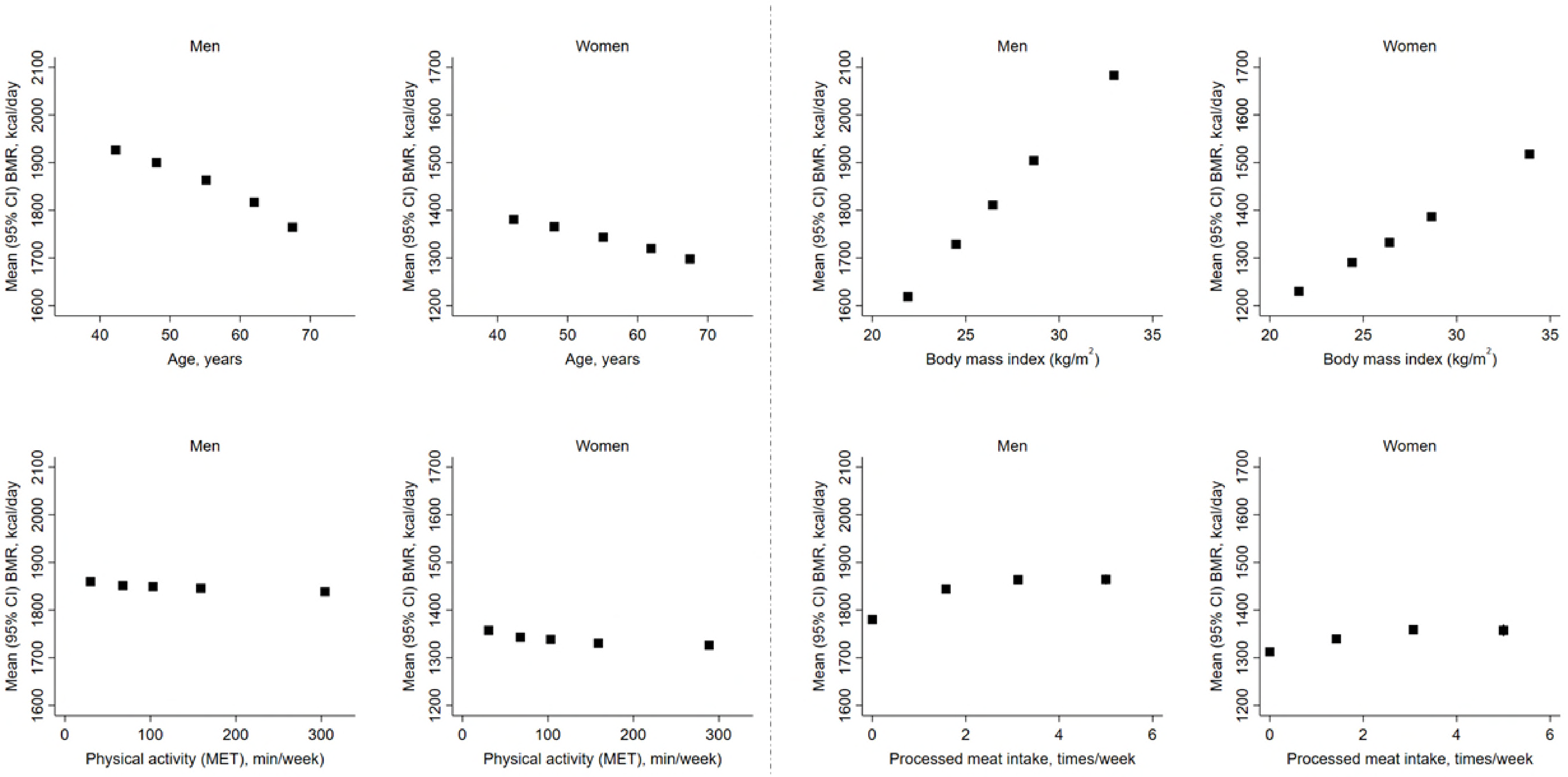


Predicted mean levels of BMR and corresponding 95% confidence intervals were derived from linear regression models, with each selected variable (age, BMI, physical activity, processed meat) modelled as categorical groups (i.e., sex-specific fifths). Marginal means were estimated and plotted at the mean value within each selected variable category. BMR=basal metabolic rate; CI=confidence intervals; MET=metabolic equivalent rate;kcal=kilocalories.

# Figure S3: Estimated BMR versus selected mediators at baseline, by sex

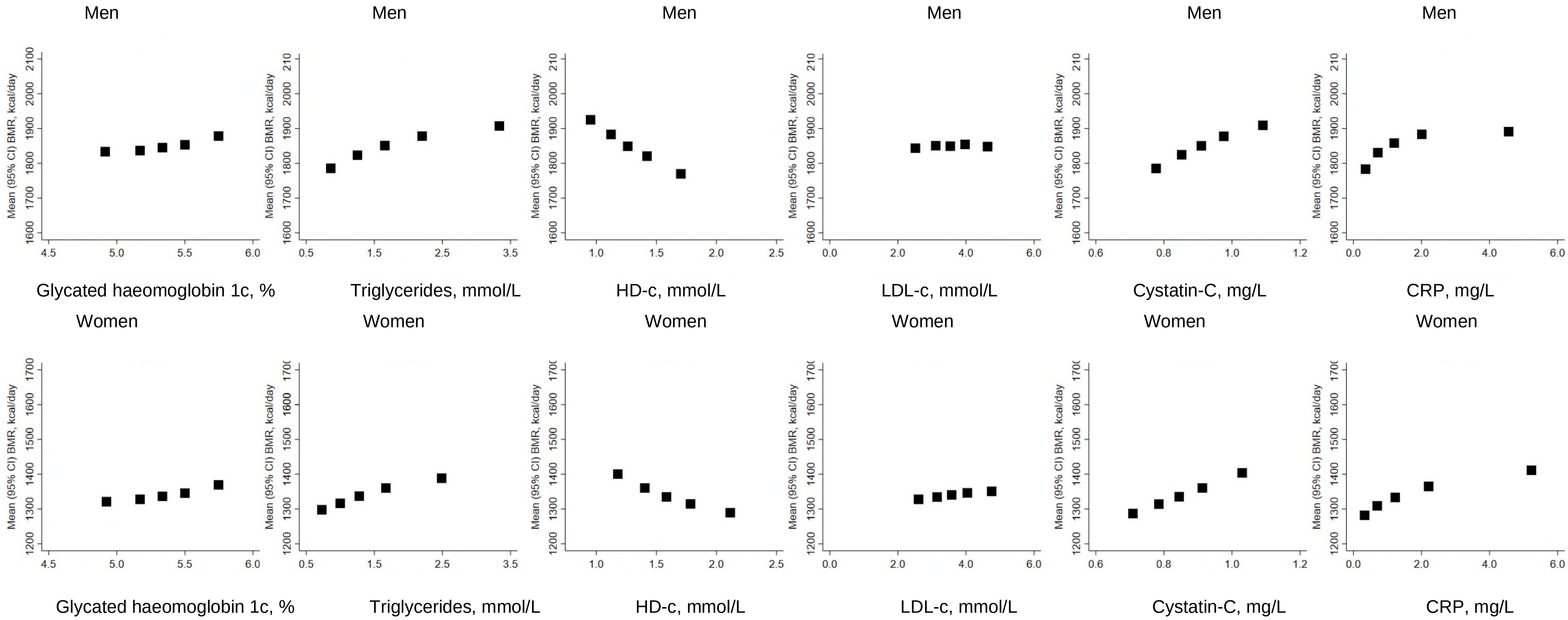


Estimates were adjusted for age. In men, the regression coefficients for the change in each mediator per SD higher BMR were: 0.14 for glycated haemoglobin, 0.19 for triglycerides, −0.07 for High-density lipoprotein cholesterol (HDL-c), 0.02 for Low-density lipoprotein cholesterol (LDL-c) , 0.01 for Cystatin-C, and 0.13 for C-reactive protein (CRP). In women, the respective coefficients were: 0.18 for glycated haemoglobin, 0.13 for triglycerides, −0.09 for HDL-c, 0.002 for LDL-c 0.02 for Cystatin-C, and 0.67 for CRP. All associations of BMR with mediators had p-values <0.001, except for LDL-c in women ( =0.323).In men, the risk of diabetes per unit increase in each mediator, adjusted for BMR were: 7.41 for HbA1c, 1.28 for triglycerides, 0.31 for HDL-c, 1.03 for LDL-c, 2.34 for Cystatin-C, and 1.03 for C-reactive protein. In women, the corresponding risks were 12.9, 1.56, 0.32, 1.25, and 2.70, respectively. All associations of potential mediators with diabetes, adjusted for BMR had p-values < 0.001 except for LDL-c in men (p = 0.240).

**Figure S4: Prevalence of diabetes by estimated BMR A-B) before recruitment; and C-D) at recruitment, stratified by sex**

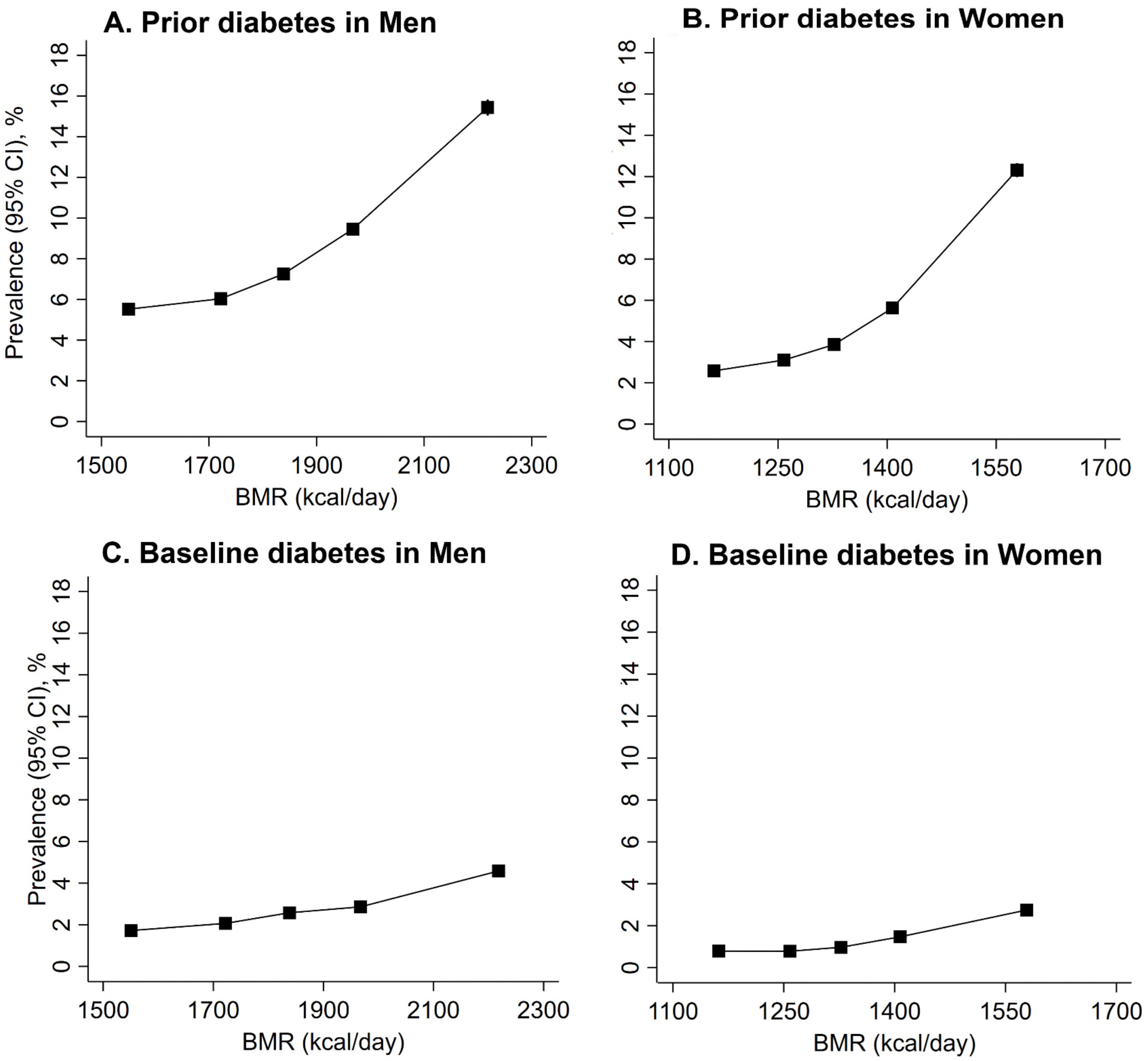


Prevalence estimates of diabetes and corresponding 95% confidence intervals were derived from marginal estimates of logistic regression models of prior baseline diabetes with BMR modelled in quintiles. Obtained estimates were plotted at the mean BMR value within each quintile. BMR=basal metabolic rate; CI=confidence intervals; kcal=kilocalories.

**Figure S5: Sensitivity analyses of the main model, now with additional adjustment for residual body mass index**

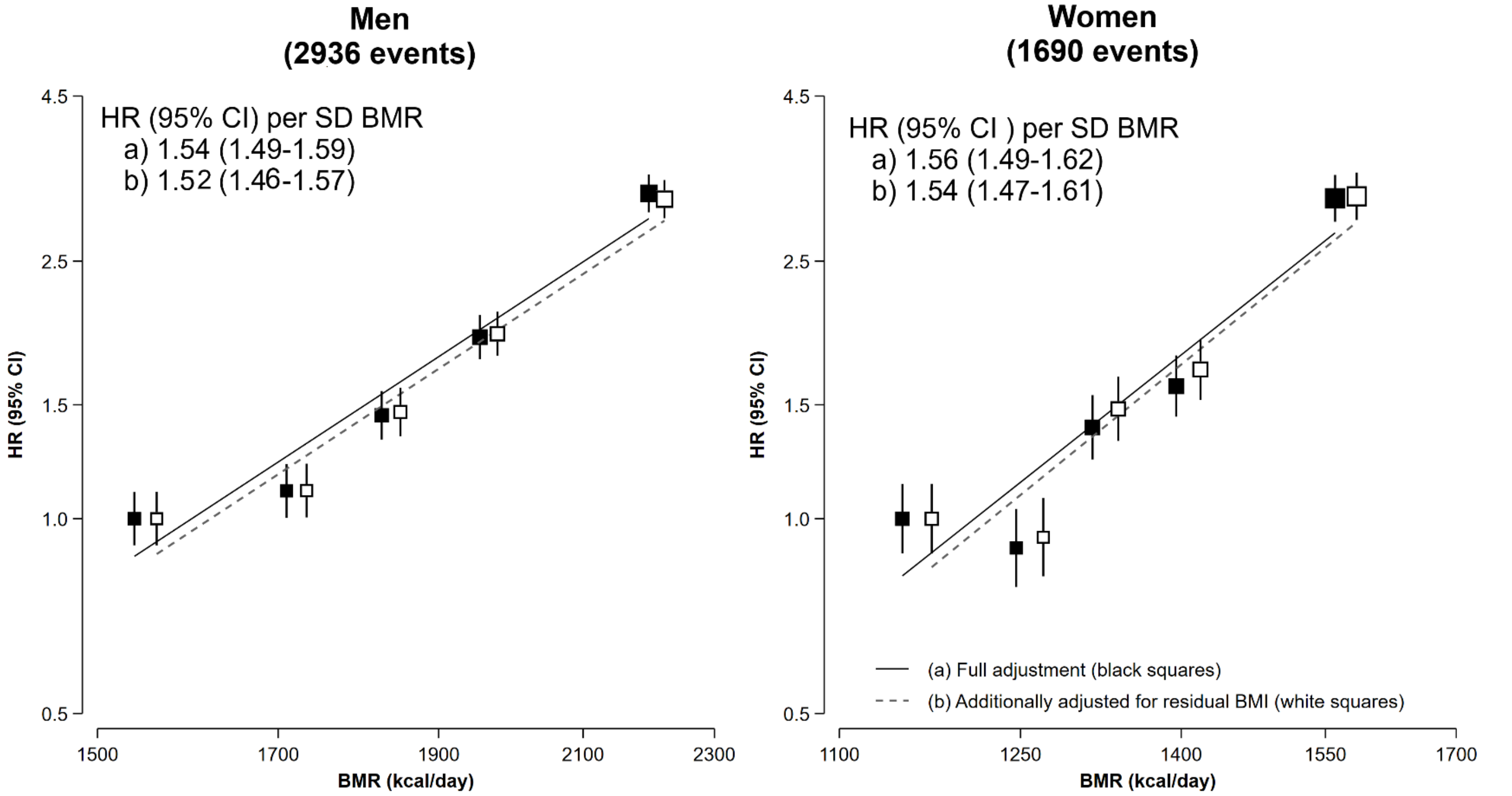


Analyses and conventions as per Figure 2. Black squares represent estimates from the main model (stratified By age-at-risk and assessment centre, and adjusted for ethnicity, Townsend Deprivation Index, physical activity, processed meat intake, alcohol consumption, and smoking). White squares represent the estimates with additional adjustment for residual BMI (or the remaining BMI after accounting for its correlation with BMR), given that the sex-specific correlations between BMI and BMR at baseline were 0.68 for men and 0.74 for women. BMR=basal metabolic rate; BMI=body mass index; CI=confidence intervals; HR=hazard ratios; kcal=kilocalories.

**Figure S6: Sensitivity analyses of association between estimated BMR and diabetes, with different exclusions**

## A) No medical exclusions

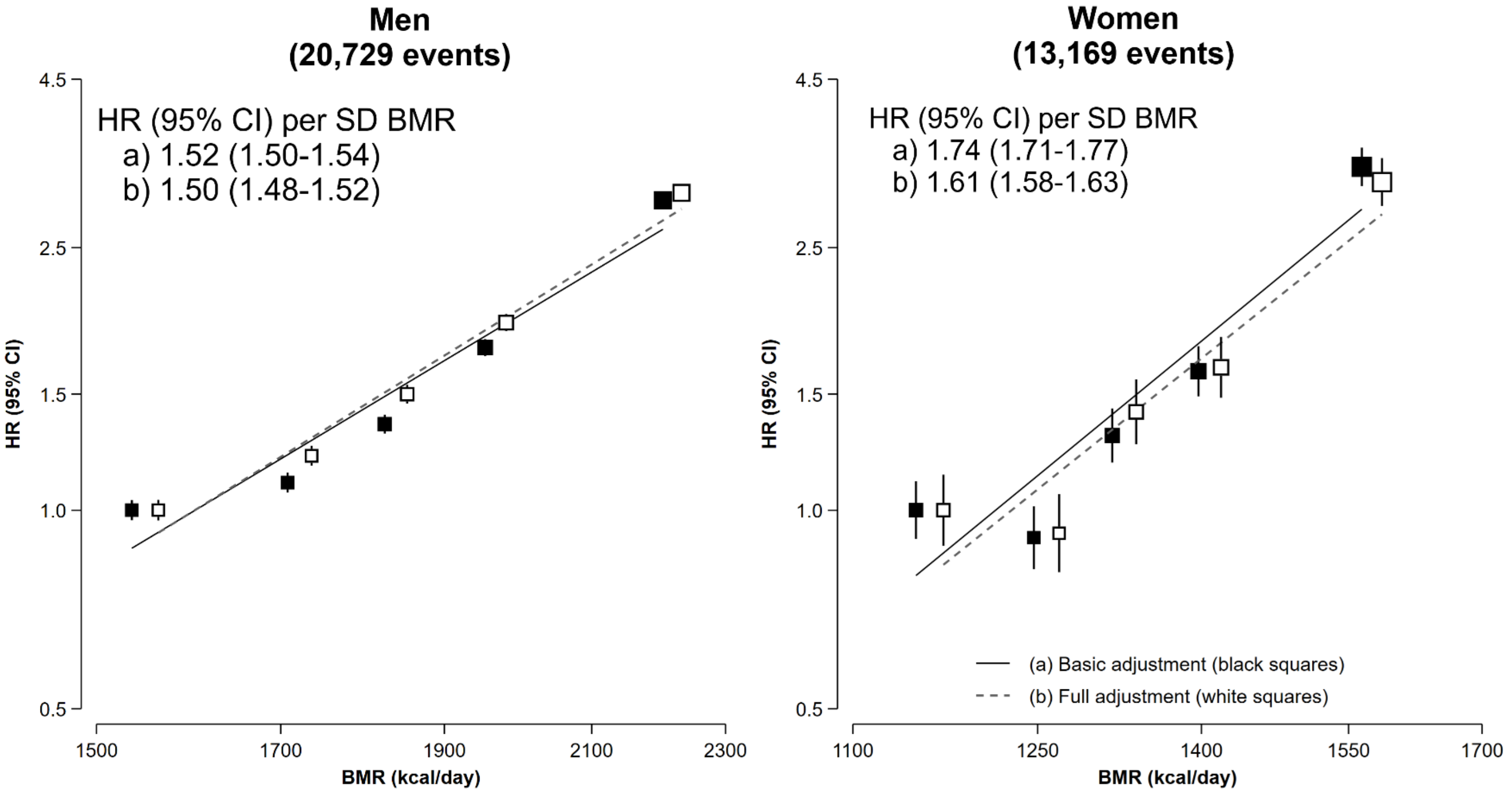


## B) Excluding pre-existing diabetes at baseline and additional adjustment for fasting

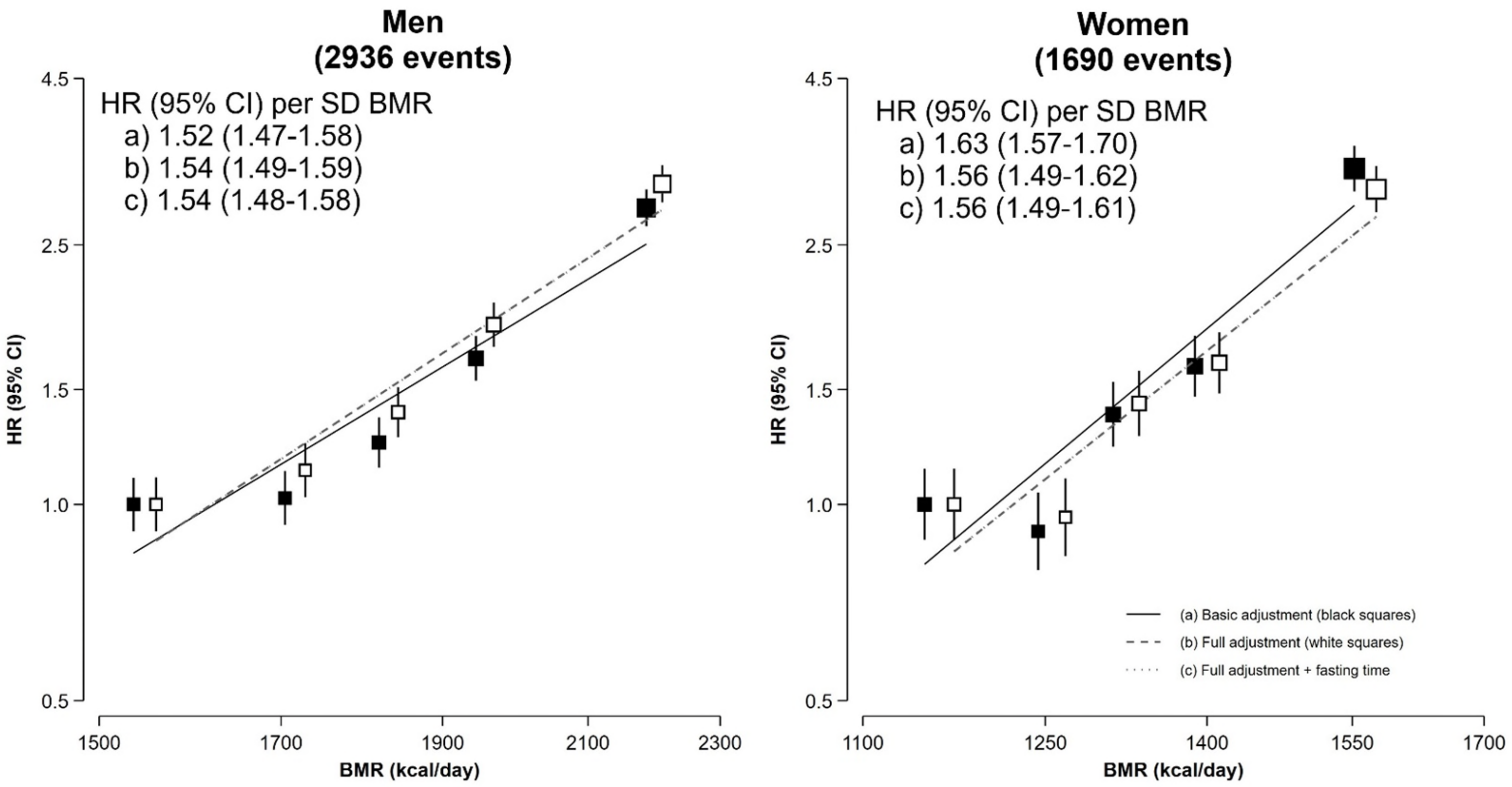

**Figure S6: continued**

**C) Excluding pre-existing diabetes and other chronic diseases at baseline**

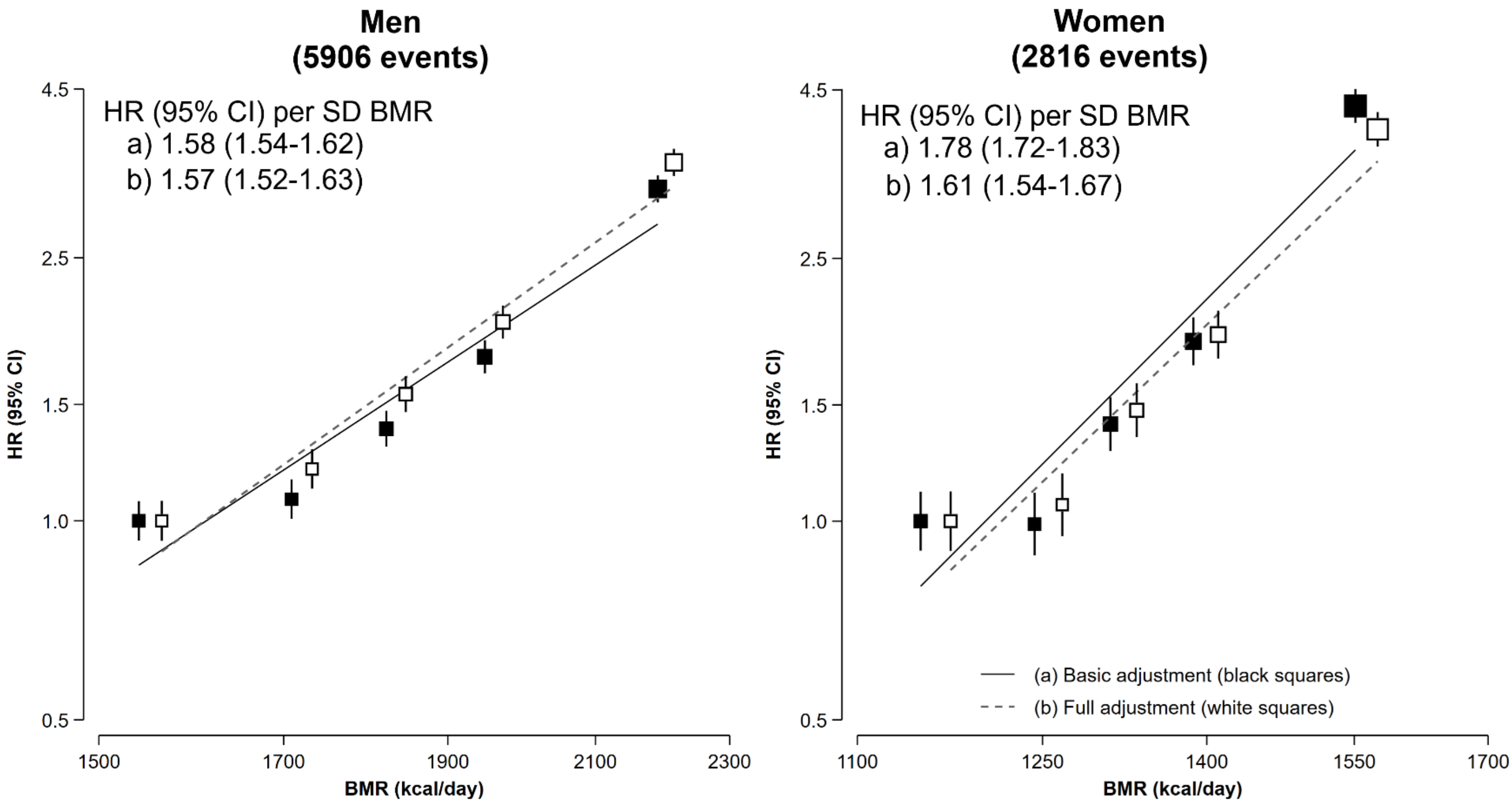


**D) Excluding any pre-existing chronic diseases at baseline and the first 5 years of follow-up**

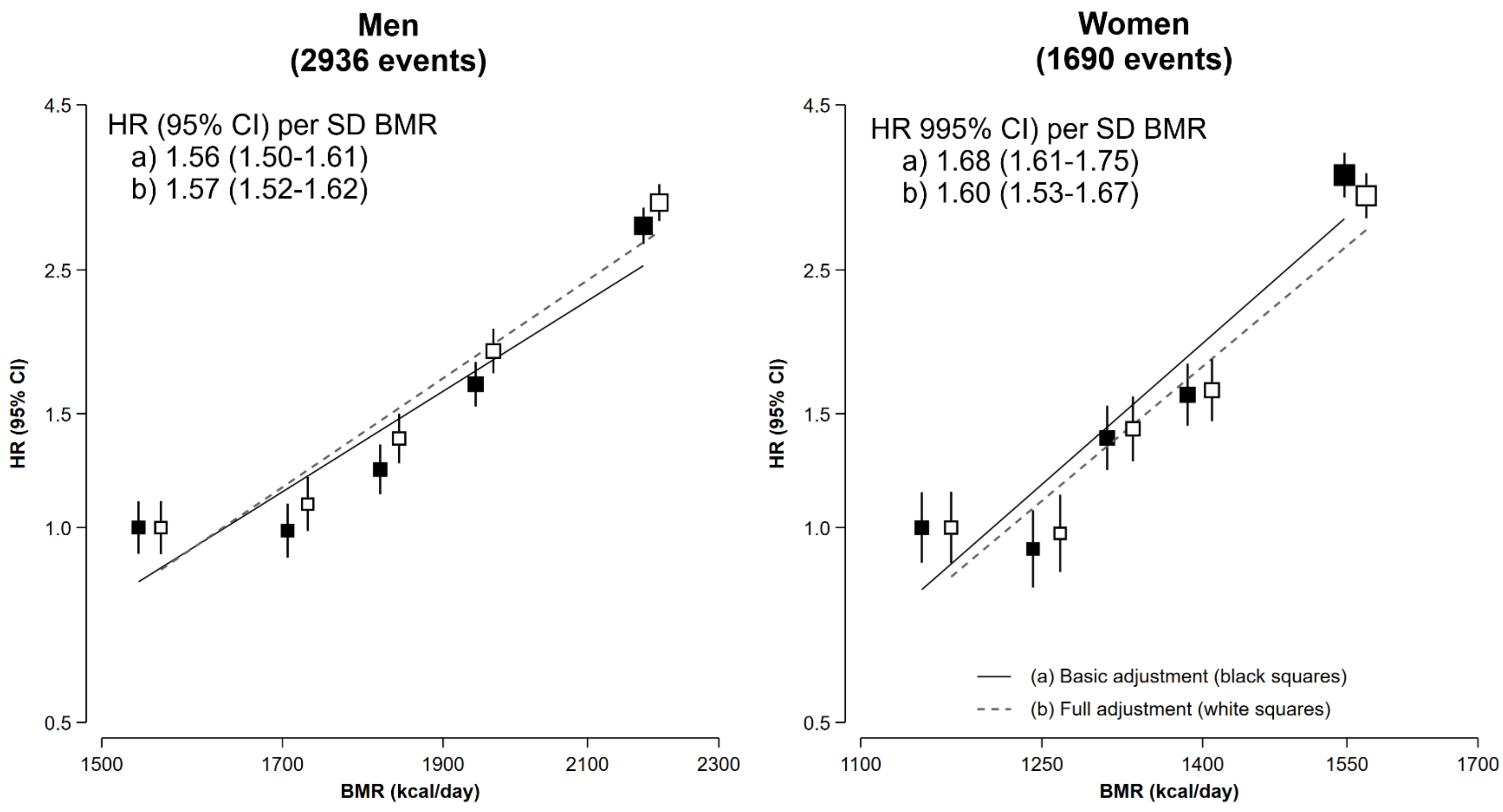

**Figure S6: continued**

**E) Excluding probable type 1 diabetes from the main outcome**

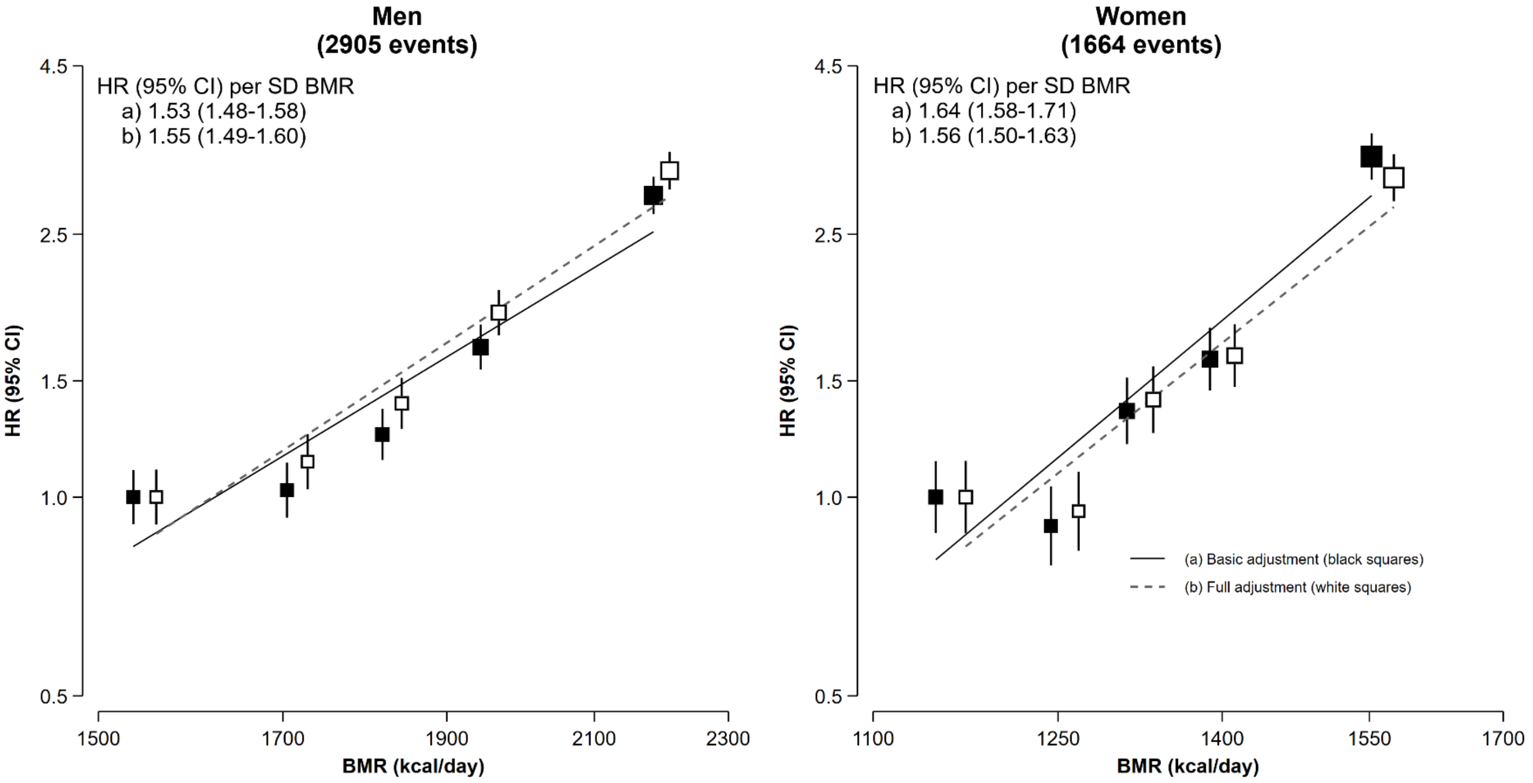


**F) Excluding those with HbA1c ≥5.7% in addition to diagnosed diabetes at baseline**

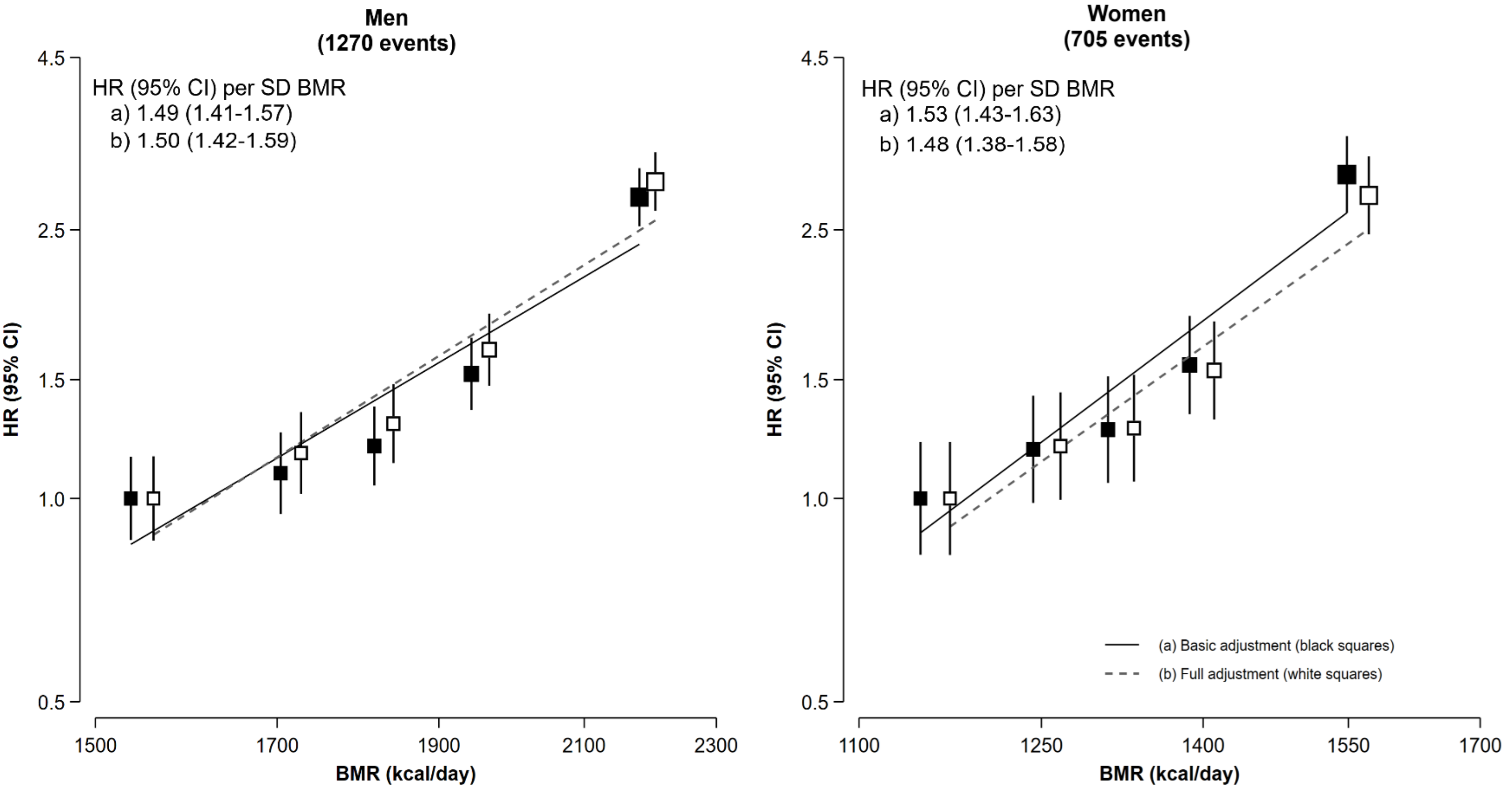


Analyses and conventions as per Figure 2, now with various criteria as specified in the graph. BMR=basal metabolic rate; CI=confidence intervals; HR=hazard ratios; kcal=kilocalories.

**Figure S7: Associations of estimated higher BMR with risk of diabetes subtypes and complications by sex, after excluding diabetes and other chornic diseases at baseline**

| | Men: No of events | Men: HR (95% CI) | Women: No of events | Women: HR (95% CI) |
|---|---|---|---|---|
| **Diabetes subtypes** | | | | |
| Type 1 | 31 | 1.14 (0.81, 1.63) | 26 | 1.25 (0.87, 1.81) |
| Type 2 | 2385 | 1.64 (1.58, 1.70) | 1274 | 1.72 (1.64, 1.80) |
| Unspecified | 520 | 1.30 (1.19, 1.41) | 390 | 1.26 (1.15, 1.38) |
| **Any diabetes** | **2936** | **1.57 (1.52, 1.63)** | **1690** | **1.61 (1.54, 1.67)** |
| **Diabetes complications** | | | | |
| None | 1802 | 1.62 (1.55, 1.69) | 1169 | 1.67 (1.59, 1.76) |
| Diabetes coma, ketoacidosis, glycaemic disturbances | 114 | 1.21 (1.00, 1.45) | 81 | 1.32 (1.08, 1.62) |
| Diabetic retinopathy | 72 | 1.35 (1.08, 1.70) | 50 | 1.33 (1.03, 1.72) |
| Other microvascular | 25 | 1.83 (1.28, 2.63) | 10 | 1.10 (0.61, 1.98) |
| Atherosclerothic diseases | 452 | 1.49 (1.36, 1.63) | 161 | 1.24 (1.07, 1.43) |
| Cardiac conditions | 331 | 1.75 (1.59, 1.94) | 152 | 1.84 (1.61, 2.10) |
| Stroke | 141 | 1.30 (1.10, 1.53) | 67 | 1.47 (1.18, 1.83) |
| **Any complication** | **1134** | **1.50 (1.42, 1.59)** | **521** | **1.45 (1.34, 1.57)** |

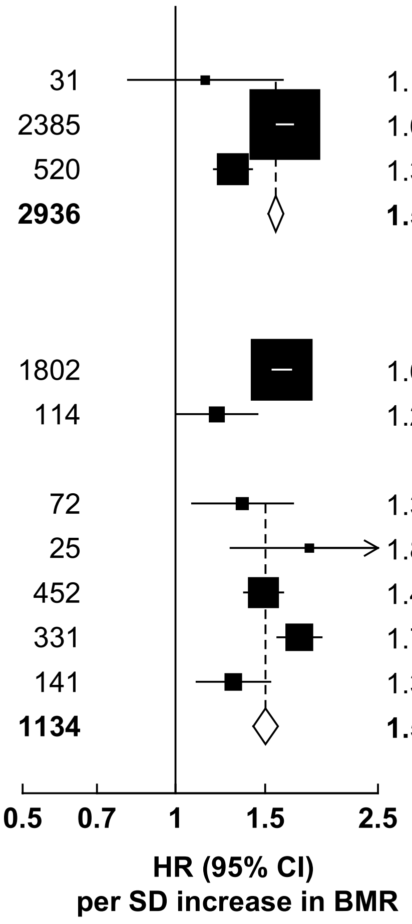


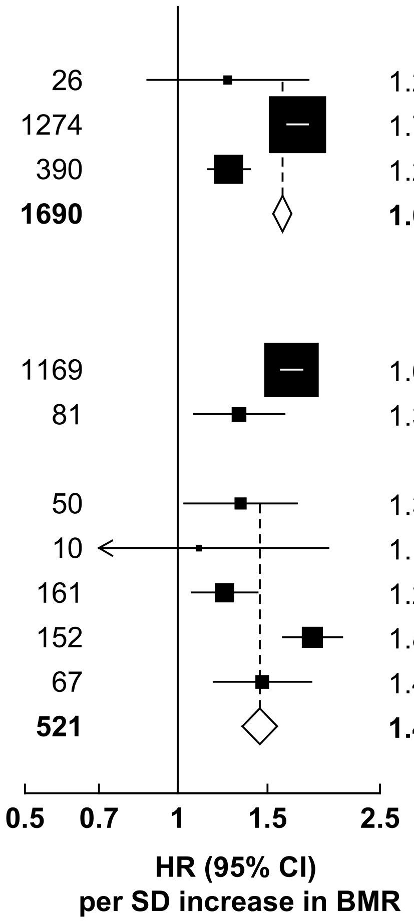


Analyses as per Figure 3, now among 129,623 men and 151,004 women, after additional exclusions of pre-exisiting medical histories at baseline.
The number of events for the diabetes outcome remained unchanged (compared to Figure 3) given that none of these cases had other prior chronic diseases following exclusions of pre-existing diabetes. BMR=Basal metabolic rate; CI=Confidence interval; HR=Hazard ratio; SD=standard deviation.

## Figure S8: Associations of estimated higher BMR with risk of diabetes subtypes and complications by sex, further excluding participants with HbA1C ≥5.7% among those undiagnosed with diabetes at baseline

| | Men: No of events | Men: HR (95% CI) | Women: No of events | Women: HR (95% CI) |
|---|---|---|---|---|
| **Diabetes subtypes** | | | | |
| Type 1 | 26 | 1.18 (0.80, 1.73) | 20 | 1.26 (0.83, 1.91) |
| Type 2 | 850 | 1.60 (1.50, 1.70) | 408 | 1.68 (1.55, 1.83) |
| Unspecified | 319 | 1.29 (1.15, 1.43) | 232 | 1.14 (1.01, 1.30) |
| **Any diabetes** | **1195** | **1.50 (1.42, 1.59)** | **660** | **1.48 (1.38, 1.58)** |
| **Diabetes complications** | | | | |
| None | 701 | 1.60 (1.50, 1.72) | 438 | 1.57 (1.44, 1.70) |
| Diabetes coma, ketoacidosis, glycaemic disturbances | 66 | 0.96 (0.74, 1.24) | 54 | 1.11 (0.85, 1.45) |
| Diabetic retinopathy | 12 | 1.16 (0.65, 2.07) | 15 | 1.45 (0.92, 2.29) |
| Other microvascular | 9 | 1.83 (1.02, 3.28) | 3 | 0.77 (0.23, 2.58) |
| Atherosclerothic diseases | 189 | 1.36 (1.18, 1.57) | 58 | 1.10 (0.85, 1.43) |
| Cardiac conditions | 156 | 1.61 (1.39, 1.87) | 67 | 1.65 (1.33, 2.04) |
| Stroke | 62 | 1.18 (0.91, 1.54) | 25 | 1.26 (0.87, 1.84) |
| **Any complication** | **494** | **1.36 (1.25, 1.48)** | **222** | **1.30 (1.15, 1.47)** |

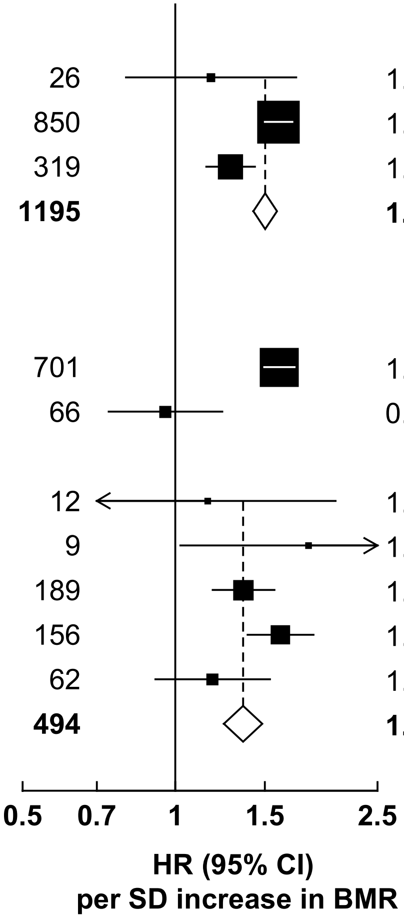


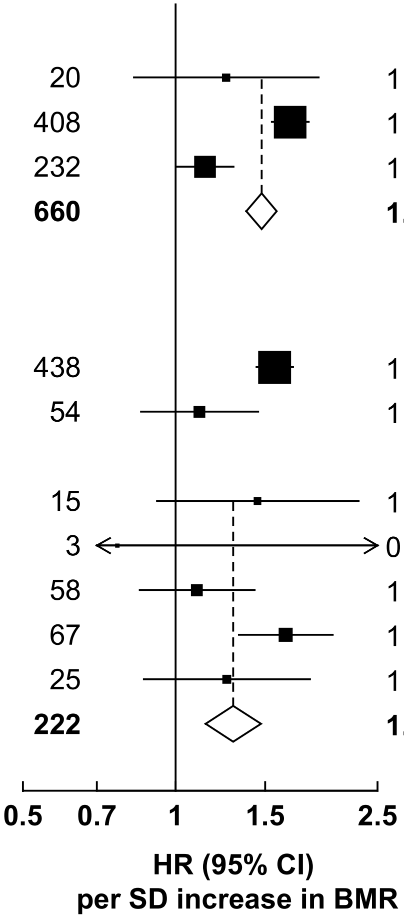


Analyses as per Figure 3, now among 131,179 men and 149,339 women, with further exclusions as specified in the graph.
BMR=Basal metabolic rate; HbA1c=glycosylated haemoglobin 1c; SD=standard deviation; HR=Hazard ratio; CI=Confidence interval.

**Figure S9: Sensitivity analyses of estimated BMR vs diabetes subtypes and complications, above BMR cut-offs of 1654 kcal/day in men and above 1219 kcal/day in women**

| | Men: No of events | Men: HR (95% CI) | Women: No of events | Women: HR (95% CI) |
|---|---|---|---|---|
| **Diabetes subtypes** | | | | |
| Type 1 | 25 | 1.14 (0.73, 1.77) | 24 | 1.16 (0.75, 1.78) |
| Type 2 | 2049 | 1.66 (1.58, 1.73) | 1114 | 1.71 (1.62, 1.80) |
| Unspecified | 420 | 1.37 (1.24, 1.53) | 306 | 1.34 (1.19, 1.50) |
| **Any diabetes** | **2494** | **1.60 (1.54, 1.67)** | **1444** | **1.62 (1.54, 1.70)** |
| **Diabetes complications** | | | | |
| None | 1555 | 1.63 (1.55, 1.71) | 1008 | 1.69 (1.60, 1.79) |
| Diabetes coma, ketoacidosis, glycaemic disturbances | 87 | 1.37 (1.09, 1.72) | 64 | 1.50 (1.18, 1.91) |
| Diabetic retinopathy | 57 | 1.33 (0.99, 1.79) | 41 | 1.26 (0.91, 1.73) |
| Other microvascular | 21 | 1.89 (1.26, 2.84) | 6 | 1.48 (0.72, 3.07) |
| Atherosclerothic diseases | 372 | 1.58 (1.42, 1.76) | 135 | 1.16 (0.96, 1.40) |
| Cardiac conditions | 293 | 1.72 (1.53, 1.93) | 134 | 1.82 (1.56, 2.12) |
| Stroke | 109 | 1.39 (1.13, 1.72) | 56 | 1.43 (1.10, 1.88) |
| **Any complication** | **939** | **1.57 (1.46, 1.68)** | **436** | **1.46 (1.33, 1.60)** |

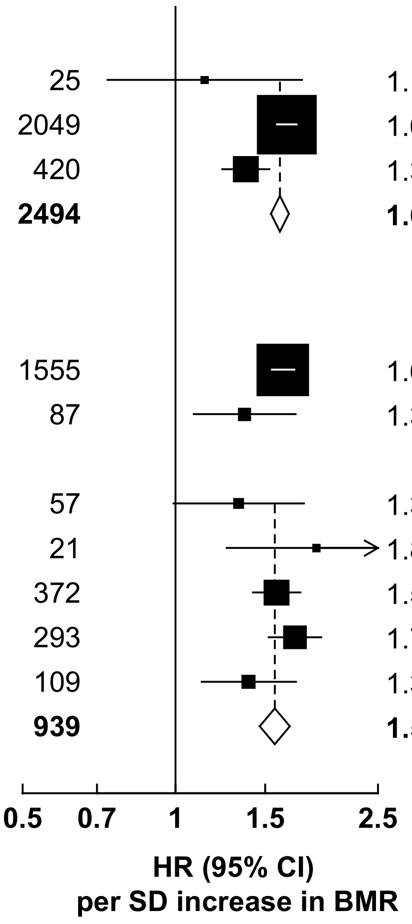


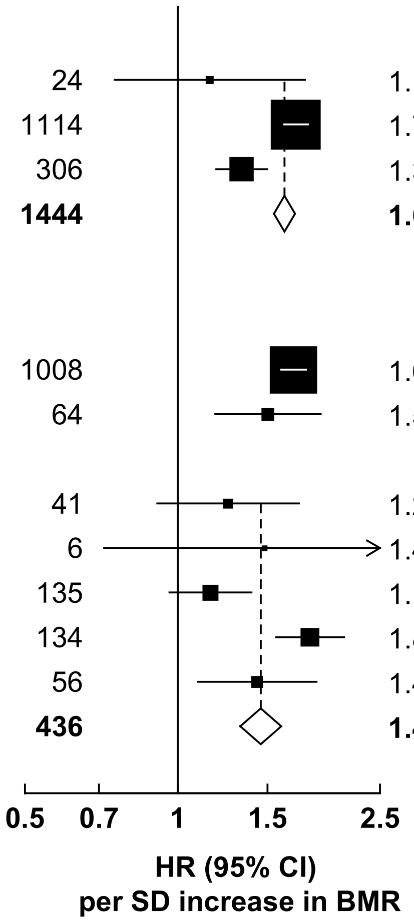


Analyses as per Figure 3, now applying a BMR threshold as indicated. The threshold corresponds to the minimum value of the second BMR quintile in both men and women, selected based on the linear associations shown in Figure 2. Analyses included 273,545 individuals (127,524 men and 146,021 women). BMR=Basal metabolic rate; kcal=kilo calories; SD=standard deviation; HR=Hazard ratio; CI=Confidence interval.

## Figure S10 : Associations of estimated BMR with diabetes given candidate vascular-metabolic mediators by sex , after additional account for residual BMI

| Mediators adjustement | Men HR (95% CI) | Men $\chi^2$ | Men %ΔHR | Women HR (95% CI) | Women $\chi^2$ | Women %ΔHR |
|---|---|---|---|---|---|---|
| BMR + confounders + residual BMI | 1.52 (1.46, 1.57) | 572.8 | - | 1.54 (1.48, 1.61) | 420.5 | - |
| .+ HbA1c | 1.52 (1.47, 1.57) | 540.8 | 0.7 | 1.48 (1.42, 1.55) | 312.5 | 9.5 |
| .+ HbA1c + Tryglicerides | 1.47 (1.41, 1.52) | 417.6 | 7.9 | 1.43 (1.36, 1.49) | 233.9 | 18.3 |
| .+ HbA1c + Tryglicerides +HDLc | 1.43 (1.37, 1.49) | 321.4 | 14.2 | 1.39 (1.33, 1.46) | 178 | 23.9 |
| .+ HbA1c + Tryglicerides +HDLc + Cystatin-C | 1.42 (1.37, 1.48) | 313.7 | 15.0 | 1.37 (1.31, 1.44) | 160.1 | 27.3 |
| .+ HbA1c + Tryglicerides +HDLc + Cystatin-C + CRP | 1.42 (1.37, 1.48) | 314 | 15.0 | 1.36 (1.29, 1.42) | 145.5 | 30.0 |

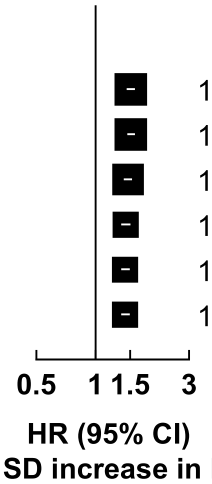


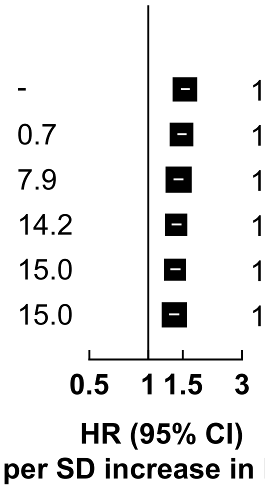


Analyses as per Figure 5, now with additional adjustment for residual BMI calculated from regressing measures of BMR on BMI.
BMI=body mass index; BMR=Basal metabolic rate; CI=Confidence interval; CRP=C-reactive protein; HbA1c=Glycated haemoglobin 1c; HDLc=High-density cholesterol; HR=Hazard ratio; SD=standard deviation; $\chi^2$=chi-squared statistic; Δ=change

**Figure S11: Regression to the mean of BMR measures between baseline and resurvey, by sex**

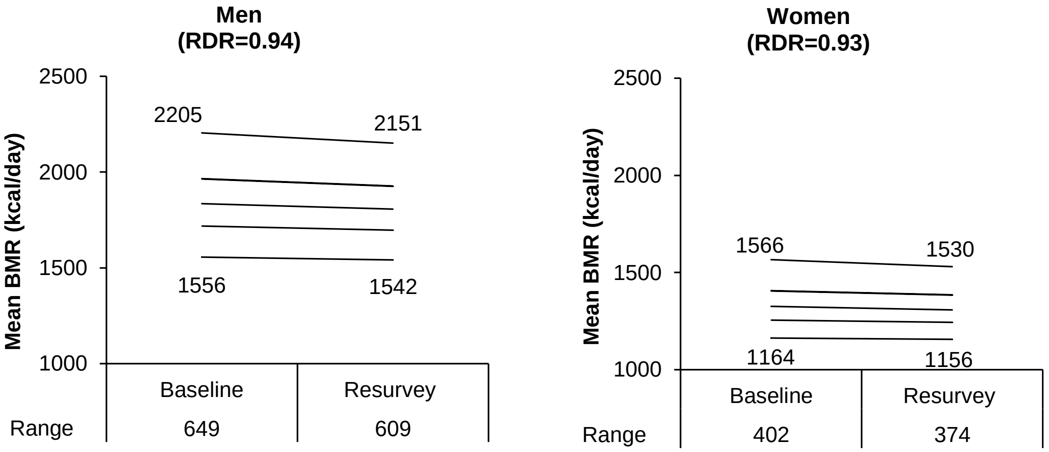


Resurvey measurements were taken on average 5 years after baseline measurements. The RDR represents the MacMahon-Peto estimate of the regression dilution calculated as the ratio of the ranges displayed at resurvey to those at baseline within fifths of the baseline markers (for example, the RDR in men was calculated as (1566-11164)/(1530-1156)=0.94). Mean BMR levels within the lower fifths are displayed at the bottom of each graph. Exclusions are as per Table 1.

STROBE Statement—Checklist of items that should be included in reports of ***cohort studies***

| | Item No | Recommendation | Page no |
|---|---|---|---|
| **Title and abstract** | 1 | (*a*) Indicate the study's design with a commonly used term in the title or the abstract | Pg. 2 |
| | | (*b*) Provide in the abstract an informative and balanced summary of what was done and what was found | Pg. 2 |
| **Introduction** | | | |
| Background/rationale | 2 | Explain the scientific background and rationale for the investigation being reported | Pg. 3, 4-5 |
| Objectives | 3 | State specific objectives, including any prespecified hypotheses | Pg. 3, 5 |
| **Methods** | | | |
| Study design | 4 | Present key elements of study design early in the paper | Pg. 5 |
| Setting | 5 | Describe the setting, locations, and relevant dates, including periods of recruitment, exposure, follow-up, and data collection | Pg. 5 |
| Participants | 6 | (*a*) Give the eligibility criteria, and the sources and methods of selection of participants. Describe methods of follow-up | Pg. 5, 6 |
| | | (*b*) For matched studies, give matching criteria and number of exposed and unexposed | n/a |
| Variables | 7 | Clearly define all outcomes, exposures, predictors, potential confounders, and effect modifiers. Give diagnostic criteria, if applicable | Pg.6-8 |
| Data sources/ measurement | 8* | For each variable of interest, give sources of data and details of methods of assessment (measurement). Describe comparability of assessment methods if there is more than one group | Pg. 6 |
| Bias | 9 | Describe any efforts to address potential sources of bias | Pg.9 Figures S6-S8 |
| Study size | 10 | Explain how the study size was arrived at | Pg.8 Figure S1 |
| Quantitative variables | 11 | Explain how quantitative variables were handled in the analyses. If applicable, describe which groupings were chosen and why | Pg. 7-8 |
| Statistical methods | 12 | (*a*) Describe all statistical methods, including those used to control for confounding | Pg. 7-9 |
| | | (*b*) Describe any methods used to examine subgroups and interactions | Pg.7-8, Figure 4 |
| | | (*c*) Explain how missing data were addressed | Pg.8, Tables S4-S5 |
| | | (*d*) If applicable, explain how loss to follow-up was addressed | Reference 15 |
| | | (*e*) Describe any sensitivity analyses | Pg.8 |
| **Results** | | | |
| Participants | 13* | (a) Report numbers of individuals at each stage of study—eg numbers potentially eligible, examined for eligibility, confirmed eligible, included in the study, completing follow-up, and analysed | Pg.9-10, Table 1, S5 |
| | | (b) Give reasons for non-participation at each stage | n/a |
| | | (c) Consider use of a flow diagram | Figure S1 |
| Descriptive data | 14* | (a) Give characteristics of study participants (eg demographic, | Pg.9, Table 1 |

| | | | |
|---|---|---|---|
| | | clinical, social) and information on exposures and potential confounders | |
| | | (b) Indicate number of participants with missing data for each variable of interest | Figure S1 |
| | | (c) Summarise follow-up time (eg, average and total amount) | Pg.10 |
| Outcome data | 15* | Report numbers of outcome events or summary measures over time | Pg.10, Table S1 |
| Main results | 16 | (*a*) Give unadjusted estimates and, if applicable, confounder-adjusted estimates and their precision (eg, 95% confidence interval). Make clear which confounders were adjusted for and why they were included | Pg.10-11, Figures 2-3, S9-S10 |
| | | (*b*) Report category boundaries when continuous variables were categorized | Pg.11, Figure S9 |
| | | (*c*) If relevant, consider translating estimates of relative risk into absolute risk for a meaningful time period | n/a |
| Other analyses | 17 | Report other analyses done—eg analyses of subgroups and interactions, and sensitivity analyses | Pg.11-12, Figures 4,S6-S10 |
| **Discussion** | | | |
| Key results | 18 | Summarise key results with reference to study objectives | Pg.13 |
| Limitations | 19 | Discuss limitations of the study, taking into account sources of potential bias or imprecision. Discuss both direction and magnitude of any potential bias | Pg.16 |
| Interpretation | 20 | Give a cautious overall interpretation of results considering objectives, limitations, multiplicity of analyses, results from similar studies, and other relevant evidence | Pg.17 |
| Generalisability | 21 | Discuss the generalisability (external validity) of the study results | Pg.17 |
| **Other information** | | | |
| Funding | 22 | Give the source of funding and the role of the funders for the present study and, if applicable, for the original study on which the present article is based | Pg.17 |

*Give information separately for exposed and unexposed groups.

**Note:** An Explanation and Elaboration article discusses each checklist item and gives methodological background and published examples of transparent reporting. The STROBE checklist is best used in conjunction with this article (freely available on the Web sites of PLoS Medicine at http://www.plosmedicine.org/, Annals of Internal Medicine at http://www.annals.org/, and Epidemiology at http://www.epidem.com/). Information on the STROBE Initiative is available at http://www.strobe-statement.org.
